# Supplementary material for: Clinical laboratory parameters associated with severe or critical novel coronavirus disease 2019 (COVID-19): A systematic review and meta-analysis
Source: PLoS One. 2020 Oct 1;15(10):e0239802. doi: 10.1371/journal.pone.0239802 (PMC7529271; doi:10.1371/journal.pone.0239802)
Supplement: S1 File — (DOCX) [file pone.0239802.s002.docx]

**Supplementary File**

**Table of Contents**

[**S1 Text. Medline search strategy** 2](#_Toc40830005)

[**S2 Text. List of eligible studies not included in meta-analysis due to overlap with included other studies** 3](#_Toc40830006)

[**S3 Table. Detailed characteristics of included studies** 5](#_Toc40830007)

[**S4 Fig. Forest plots and Leave-one-out analysis for all laboratory parameters** 22](#_Toc40830008)

[**S5 Table. Risk of bias assessment using NIH Quality Assessment Tool** 51](#_Toc40830009)

[**S6 Table. Sensitivity analysis reporting mean differences obtained by estimating the sample mean and variance from the reported median and interquartile range (or range)** 54](#_Toc40830010)

[**References for supplementary file** 56](#_Toc40830011)

# **S1 Text. Medline search strategy**

Database: Ovid MEDLINE(R) and Epub Ahead of Print, In-Process & Other Non-Indexed Citations and Daily <1946 to April 17, 2020>

Date of search: April 18, 2020, 22:52 hrs CET

Search Strategy:

--------------------------------------------------------------------------------

1 SARS-CoV-2.ab,ti,kw. (1396)

2 COVID-19.ab,ti,kw. (4280)

3 2019 nCoV.ab,ti,kw. (589)

4 Novel coronavirus.ab,ti,kw. (1506)

5 Wuhan coronavirus.ab,ti,kw. (15)

6 Wuhan pneumonia.ab,ti,kw. (12)

7 2019 coronavirus.ab,ti,kw. (52)

8 coronavirus disease-19.ab,ti,kw. (51)

9 SARS2.ab,ti,kw. (7)

10 1 or 2 or 3 or 4 or 5 or 6 or 7 or 8 or 9 (5613)

11 exp clinical laboratory techniques/ or exp clinical chemistry tests/ or exp microbiological techniques/ or exp hematologic tests/ or exp immunologic tests/ (2567586)

12 ((white adj2 cell*) or neutrophil* or lymphocyt* or monocyt* or platelet* or thrombocyt* or hemoglobin or CD* or bilirubin or ALT or AST or protein or globulin or albumin or urea or creat* or glucose or troponin or cholinesterase or cystatin or dehydrogenase or interleukin or erythrocye or procalcitonin or ferritin or prothromb* or D-dimer or sodium or potassium or calcium or chloride).ab,ti,kw. (5599430)

13 (blood test or laboratory test).ab,ti,kw. (14239)

14 clinical.ab,ti,kw. (3458054)

15 11 or 12 or 13 or 14 (9864764)

16 10 and 15 (1516)

17 limit 16 to yr="2019 -Current" (1318)

***************************

# **S2 Text. List of eligible studies not included in meta-analysis due to overlap with included other studies**

1. Cai Q, Huang D, Yu H, Zhu Z, Xia Z, Su Y, et al. Characteristics of Liver Tests in COVID-19 Patients. Journal of hepatology. 2020. Matched to Cai et al. and Zeng et al.

2. Chen C, Yan JT, Zhou N, Zhao JP, Wang DW. Analysis of myocardial injury in patients with COVID-19 and association between concomitant cardiovascular diseases and severity of COVID-19. [Chinese]. Zhonghua xin xue guan bing za zhi 2020;48:E008 Matched to Qin et al. and Chen G et al.

3. Chen L, Liu HG, Liu W, et al. [Analysis of clinical features of 29 patients with 2019 novel coronavirus pneumonia]. Chung-Hua Chieh Ho Ho Hu Hsi Tsa Chih Chinese Journal of Tuberculosis & Respiratory Diseases 2020;43(3):203-08 Matched to Qin et al. and Chen G et al.

4. Gao Y, Li T, Han M, et al. Diagnostic Utility of Clinical Laboratory Data Determinations for Patients with the Severe COVID-19. Journal of Medical Virology 2020;17:17 Matched to Wu Jian et al.

5. Gong J, Dong H, Xia SQ, et al. Correlation Analysis Between Disease Severity and Inflammation-related Parameters in Patients with COVID-19 Pneumonia. medRxiv 2020:2020.02.25.20025643 doi: 10.1101/2020.02.25.20025643[published Online First: Epub Date]|. Matched to Qin et al. and Chen G et al.

6. Han H, Yang L, Liu R, et al. Prominent changes in blood coagulation of patients with SARS-CoV-2 infection. Clinical Chemistry & Laboratory Medicine 2020;16:16 Matched to Luo et al. Han Yi et al.

7. Han H, Xie L, Liu R, Yang J, Liu F, Wu K, et al. Analysis of heart injury laboratory parameters in 273 COVID-19 patients in one hospital in Wuhan, China. Journal of medical virology. 2020. Matched to Luo et al. Han Yi et al.

8. Huang H, Cai S, Li Y, et al. Prognostic factors for COVID-19 pneumonia progression to severe symptom based on the earlier clinical features: a retrospective analysis. medRxiv 2020:2020.03.28.20045989 doi: 10.1101/2020.03.28.20045989[published Online First: Epub Date]|. Matched to Gong Jiao et al.

9. Hui H, Zhang Y, Yang X, et al. Clinical and radiographic features of cardiac injury in patients with 2019 novel coronavirus pneumonia. medRxiv 2020:2020.02.24.20027052 doi: 10.1101/2020.02.24.20027052[published Online First: Epub Date]|. Matched to Zhao et al.

10. Lei l, Jian-ya G. Clinical characteristics of 51 patients discharged from hospital with COVID-19 in Chongqing，China. medRxiv 2020:2020.02.20.20025536 doi: 10.1101/2020.02.20.20025536[published Online First: Epub Date]|. Matched to Wan Suxin et al.

11. Li K, Wu J, Wu F, et al. The Clinical and Chest CT Features Associated with Severe and Critical COVID-19 Pneumonia. Investigative Radiology 2020;29:29 Matched to Wan Suxin et al.

12. Li X, Xu S, Yu M, Wang K, Tao Y, Zhou Y, et al. Risk factors for severity and mortality in adult COVID-19 inpatients in Wuhan. The Journal of allergy and clinical immunology. 2020. ]|. Matched to Qin et al. and Chen G et al.

13. Liu J, Li S, Liu J, et al. Longitudinal characteristics of lymphocyte responses and cytokine profiles in the peripheral blood of SARS-CoV-2 infected patients. medRxiv 2020:2020.02.16.20023671 doi: 10.1101/2020.02.16.20023671[published Online First: Epub Date]|. Matched to Wang et al. and Liu Tao et al.

14. Liu Y, Yang Y, Zhang C, et al. Clinical and biochemical indexes from 2019-nCoV infected patients linked to viral loads and lung injury. Science China. Life sciences 2020;63(3):364-74 Matched to Cai Q et al. and Zeng et al.

15. Peng YD, Meng K, Guan HQ, et al. Clinical characteristics and outcomes of 112 cardiovascular disease patients infected by 2019-nCoV. [Chinese]. Zhonghua xin xue guan bing za zhi 2020;48:E004 Matched to Qin et al. and Chen G et al.

16. Qi D, Yan X, Tang X, et al. Epidemiological and clinical features of 2019-nCoV acute respiratory disease cases in Chongqing municipality, China: a retrospective, descriptive, multiple-center study. medRxiv 2020:2020.03.01.20029397 doi: 10.1101/2020.03.01.20029397[published Online First: Epub Date]|. Matched to Wan Suxin et al., Yuan et al. and Zhang Hui et al.

17. Shi Y, Tan M, Chen X, et al. Immunopathological characteristics of coronavirus disease 2019 cases in Guangzhou, China. medRxiv 2020:2020.03.12.20034736 doi: 10.1101/2020.03.12.20034736[published Online First: Epub Date]|. Matched to Gong et al.

18. Wan S, Yi Q, Fan S, et al. Characteristics of lymphocyte subsets and cytokines in peripheral blood of 123 hospitalized patients with 2019 novel coronavirus pneumonia (NCP). medRxiv 2020:2020.02.10.20021832 doi: 10.1101/2020.02.10.20021832[published Online First: Epub Date]|. Matched to Wan Suxin et al.

19. Wang D, Hu B, Hu C, et al. Clinical Characteristics of 138 Hospitalized Patients with 2019 Novel Coronavirus-Infected Pneumonia in Wuhan, China. JAMA - Journal of the American Medical Association 2020;323(11):1061-69 Matched to Zhang G et al.

20. Wang F, Nie J, Wang H, et al. Characteristics of peripheral lymphocyte subset alteration in COVID-19 pneumonia. Journal of Infectious Diseases 2020;30:30 Matched to Zhang G et al.

21. Wu J, Liu J, Zhao X, et al. Clinical Characteristics of Imported Cases of COVID-19 in Jiangsu Province: A Multicenter Descriptive Study. Clinical Infectious Diseases 2020;29:29 Matched to Wu Jian et al.

22. Yang J-K, Jin J-M, Liu S, Bai P, He W, Wu F, et al. Blood glucose is a representative of the clustered indicators of multi-organ injury for predicting mortality of COVID-19 in Wuhan, China. medRxiv. 2020:2020.04.08.20058040. Matched to Wang et al. and Liu Tao et al.

# **S3 Table. Detailed characteristics of included studies**

| **Study** | **Study characteristics** | **Patient characteristics** | **^a^Risk of Bias** |
| --- | --- | --- | --- |
| Cai et al.[1] | **Hospital(s):** Third people's Hospital of Shenzhen  **Location(s):** Shenzhen  **Study period:** 11 Jan 2020 - 6 Feb 2020  **Aim:** To study the clinical characteristics of affected Covid-19 patients outside the epicentre of Hubei province.  **Study design:** Retrospective cohort study  **Sample size:** 298  **Study COVID-19 case definition:** Laboratory confirmed cases (RT-PCR)  **COVID-19 severity classification:** Severe vs non-severe. Based on American Thoracic Society and Infectious Disease Society of America guidelines for community acquired pneumonia.  **Timing of classification of severity:** On admission  **Timing of blood sample collection:** On admission | **Number of mild COVID-19 cases:** 240  **Number of severe/critical COVID-19 cases:** 58  **^b^Age:** 47 (33-61) years, (mild: 40 [31-56] years, severe/critical: 64 [56-66] years)  **Males:** 149/298 (50.0%), (mild: 111/240 [46.3%], severe/critical: 33/58 [56.9%])  **Hypertension:** 38/298 (12.8%)  **Diabetes:** 19/298 (6.4%)  **Cancer:** 4/298 (1.4%) | **Low** |
| Cao Min et al.[2] | **Hospital(s):** Shanghai Public Health Clinical Centre  **Location(s):** Shanghai, China  **Study period:** x Jan 2020 - x Feb 2020  **Aim:** To describe clinical features of COVID-19 in Shanghai and to compare clinical features between non-severe and severe cases  **Study design:** Retrospective cohort study  **Sample size:** 198  **Study COVID-19 case definition:** Laboratory confirmed cases (RT-PCR)  **COVID-19 severity classification:** Mild, Moderate, Severe, Critical based on criteria by CNHC  **Timing of classification of severity:** On admission  **Timing of blood sample collection:** On admission | **Number of mild COVID-19 cases:** 179  **Number of severe/critical COVID-19 cases:** 19  **^c^Age:** 50.1 (16.3) years, (mild: 48.6 [15.6] years, severe/critical: 63.7 [16.8] years)  **Males:** 101/198 (51.0%), (mild: 84/179 [46.9%], severe/critical: 17/19 [89.5%])  **Hypertension:** 42/198 (21.2%), (mild: 36/179 [20.1%], severe/critical: 6 [31.6%])  **Diabetes:** 15/198 (7.6%), (mild: 13/179 [7.3%], severe/critical: 2/19 [10.5%])  **Cancer:** 4/198 (2.0%), (mild: 4/179[2.2%]), severe/critical: 0 [0.0%)] | **Low** |
| Cao Weiliang et al.[3] | **Hospital(s):** Xiangyang No.1 Hospital  **Location(s):** Xiangyang, China  **Study period:** 1 Jan 2020 - 16 Feb 2020  **Aim:** To show the infectious characteristics of COVID-19 by analysing the lab confirmed cases and describe the risk factors for prognosis of severe Covid cases  **Study design:** Retrospective cohort study  **Sample size:** 128  **Study COVID-19 case definition:** Laboratory confirmed cases (RT-PCR)  **COVID-19 severity classification:** Non-severe, Severe (Criteria NR)  **Timing of classification of severity:** On admission  **Timing of blood sample collection:** On admission | **Number of non-severe COVID-19 cases:** 107  **Number of severe/critical COVID-19 cases:** 21  **Age:** >65 years= 24/128 (18.8%), (non-severe: >65 yrs = 19/107 [17.8%], severe: >65 yrs = 5/21 [23.8%])  **Males:** 60/128 (46.9%), (mild: 48/107 [44.9%], severe: 12/21 [57.1%])  **Hypertension:** NR**, Diabetes:** NR, **Cancer:** NR | **High** |
| Chen Dong et al.[4] | **Hospital(s):** Hospitals in Wenzhou (Wenzhou Central Hospital, 6th People’s Hospital of Wenzhou)  **Location(s):** Wenzhou, Zhejiang Province, China  **Study period:** 11 Jan 2020 - 15 Feb 2020  **Aim:** To determine the relationship between hypokalemia and clinical features, the underlying causes and clinical implications of hypokalemia in Covid-19 cases.  **Study design:** Retrospective cohort study  **Sample size:** 175  **Study COVID-19 case definition:** Laboratory confirmed cases (RT-PCR)  **COVID-19 severity classification:** Mild, moderate, severe, critical based on criteria by CNHC  **Timing of classification of severity:** NR  **Timing of blood sample collection:** NR | **Number of moderate/mild COVID-19 cases:** 135  **Number of severe/critical COVID-19 cases:** 40  **^b^Age:** 46(34-54) years  **Males:** 83/175 (47.4%)  **Hypertension:** 28/175 (16.0%)  **Diabetes:** 12/175 (6.8%)  **Cancer:** NR | **Low** |
| Chen Guang et al.[5] | **Hospital(s):** Tongji Hospital  **Location(s):** Wuhan, China  **Study period:** late Dec 2019 – 27 Jan 2020  **Aim:** This research aimed to delineate and compare the immunologic features of severe and moderate Covid-19.  **Study design:** Retrospective cohort study  **Sample size:** 21  **Study COVID-19 case definition:** Laboratory confirmed (RT-PCR)  **COVID-19 severity classification:** Mild, moderate, severe, critical based on criteria by CNHC  **Timing of classification of severity:** On admission  **Timing of blood sample collection:** On admission | **Number of mild COVID-19 cases:** 10  **Number of severe/critical COVID-19 cases:** 11  **^b^Age:** 56 (50-65) years, (mild: 52 [42.8-56] years, severe/critical: 61 [56.5-66] years)  **Males:** 17/21 (81.0%), (mild: 7/10 [70%], severe/critical: 10/11 [90.9%])  **Hypertension:** 5/21 (23.8%), (mild: 1/10 [10%], severe/critical: 4/11 [36.4%])  **Diabetes:** 3/21 (14.3%), (mild: 1/10 [10%], severe/critical: 2/11 [18.2%]), **Cancer:** NR | **Medium** |
| Chen Meizhu et al. [6] | **Hospital(s):** Fifth Affiliated Hospital of Sun Yat-sen University  **Location(s):** Zhuhai, China  **Study period:** 17 Jan 2020 - 10 March 2020  **Aim:** To put forward prediction index of severe COVID-19 patients, principles of early intervention and methylprednisolone usages in COVID-19 patients.  **Study design:** Retrospective cohort study  **Sample size:** 97  **Study COVID-19 case definition:** Laboratory confirmed cases (RT-PCR)  **COVID-19 severity classification:** Non-severe and severe based on criteria by CNHC  **Timing of classification of severity:** On admission and in wards  **Timing of blood sample collection:** On admission and at different stages | **Number of moderate/mild COVID-19 cases:** 71  **Number of severe/critical COVID-19 cases:** 26  **^d^Age:** 47.5(15-80) years, (mild: 44 [15-75] years, severe: 56.8 [32-80] years]  **Males:** 42/97 (43.3%), (mild:27/71[38%], severe: 15/26 [57.7%])  **Hypertension:** 16/97 (16.5%), (mild:10/71 [14.1%], severe: 6/26 [23.1%])  **Diabetes:** 6/97 (6.2%), (mild: 2/71 [2.8%], severe: 4/26 [15.4%])  **Cancer:** 6/97 (6.2%), (mild: 4/71 [5.6%], severe: 2/26 [7.6%]) | **Low** |
| Dai et al.[7] | **Hospital(s):** Hospitals in Hunan Province  **Location(s):** Hunan province  **Study period:** 21 Jan 2020 - 13 Feb 2020  **Aim:** To analyze the clinical characteristics of novel coronavirus pneumonia in Hunan province and provide scientific evidence for the discovery, treatment and prevention of NCP cases.  **Study design:** Retrospective cohort study  **Sample size:** 918  **Study COVID-19 case definition:** Laboratory and clinical confirmation based on criteria by CNHC  **COVID-19 severity classification:** Mild, moderate, severe, critical based on criteria by CNHC  **Timing of classification of severity:** NR  **Timing of blood sample collection:** NR | **Number of mild COVID-19 cases:** 77  **Number of severe/critical COVID-19 cases:** 841  **^c^Age:** 44.73 (16.0) years  **Males:** 479/918 (52.18%)  **Hypertension:** NR  **Diabetes:** NR  **Cancer:** NR | **High** |
| Fang et al.[8] | **Hospital(s):** Anhui Provincial Hospital  **Location(s):** Anhui province  **Study period:** 22 Jan 2020 - 18 Feb 2020  **Aim:** To retrospectively analyze the clinical characteristics of patients with new coronavirus infection pneumonia (COVID-19), summarize the treatment experience, and provide diagnosis and treatment reference for frontline clinicians.  **Study design:** Retrospective study  **Sample size:** 79  **Study COVID-19 case definition:** Laboratory confirmed cases (RT-PCR)  **COVID-19 severity classification:** Mild, moderate, severe, critical based on criteria by CNHC  **Timing of classification of severity:** NR  **Timing of blood sample collection:** 48 hours of admission for mild cases, and 48 hours of admission into ICU for severe/critical cases | **Number of mild COVID-19 cases:** 55  **Number of severe/critical COVID-19 cases:** 24  **^c^Age:** 45.1 (16.6) years, (mild: 39.9 [14.9] years, severe/critical: 56.7 [14.4] years)  **Males:** 45/79 (57.0%), (mild: 27/55 [49%], severe/critical: 18/24 [75%])  **Hypertension:** 16/79 (20.3%), (mild: 5/55 [9.1%], severe/critical: 11/24 [45.8%])  **Diabetes:** 8/79 (14.5%), (mild: 4/55 [7.3%], severe/critical: 4/24 [16.7%])  **Cancer:** 1/79 (1.3%), (mild: 1/55 [1.8%]), severe/critical: 0 [0.0%)] | **Low** |
| Gong et al.[9] | **Hospital(s):** Guangzhou Eighth People's Hospital, Zhongnan Hospital of Wuhan University and the Third Affiliated Hospital of Sun Yat-sen University, but 189 used in the analysis come only from Guangzhou Eighth People's Hospital  **Location(s):** Guangzhou  **Study period:** 20 Jan 2020 – 2 March 2020  **Aim:** To develop an effective risk nomogram to distinguish individuals with severe COVID-19 from non-severe COVID-19.  **Study design:** Retrospective cohort study  **Sample size:** 189  **Study COVID-19 case definition:** Laboratory confirmed cases (RT-PCR)  **COVID-19 severity classification:** Non-severe vs severe. For severe, at least one of the following conditions should be met: (1) Shortness of breath, Respiratory rate (RR) ≥30times/min, (2) Arterial oxygen saturation (Resting status) ≤93%, or (3) the ratio of Partial pressure of oxygen to Fraction of inspiration O-2（PaO-2/ FiO-2）≤300mmHg.  **Timing of classification of severity:** Non-severe cases all classified on admission. Severe cases classified during follow-up in the wards if they developed criteria. Median time to develop severe COVID-19 unspecified  **Timing of blood sample collection:** On admission | **Number of non-severe COVID-19 cases:** 161  **Number of severe/critical COVID-19 cases:** 28  **^b^Age:** 49.0 (35.0-63.0) years, (mild: 45.0 [33.0-62.0] years, severe: 63.5 [54.5-72.0] years)  **Males:** 88/189 (46.6%), (mild: 72/161 [44.7%], severe: 16/28 [57.1%])  **Proportion with at least one severe disease (Hypertension, Diabetes, Cardiovascular disease, Chronic Respiratory Disease, Tuberculosis):** 55/189 (29.1%), (mild: 41/161 [25.5%], severe: 14/28 [50.0%]) | **Low** |
| Goyal et al. [10] | **Hospital(s):** New York-Presbyterian Hospital’s Weill Cornell Medical Center and Lower Manhattan Hospitals  **Location(s):** New York, USA  **Study period:** 5 March 2020 – 27 March 2020  **Aim:** To characterize the first 393 consecutive patients with Covid-19 who were admitted to two hospitals in New York City.  **Study design:** Case Series  **Sample size:** 393  **Study COVID-19 case definition:** Laboratory confirmed cases (RT-PCR)  **COVID-19 severity classification:** Respiratory failure leading to invasive mechanical ventilation vs. no invasive mechanical ventilation  **Timing of classification of severity:** In the wards.  **Timing of blood sample collection:** 48 hours within admission | **Number of non-severe COVID-19 cases:** 263  **Number of severe/critical COVID-19 cases:** 130  **^b^Age:** 62.2 (48.6-73.7) years, (mild: 61.5 [47.0-75.0] years, severe: 64.5 [51.7-73.6] years)  **Males:** 238/393 (60.6%), (mild: 146/263 [55.5%], severe: 92/130 [70.8%])  **Hypertension:** 197/393 (50.1%), (mild: 127/263 [48.3%], severe/critical: 70/130 [53.8%])  **Diabetes:** 99/393 (25.2%), (mild: 63/263 [24%], severe/critical: 36/130 [27.7%])  **Cancer:** 23/393 (5.9%), (mild: 13/263 [4.9%]), severe/critical: 10/130 [7.7%)] | **Low** |
| Guan et al.[11] | **Hospital(s):** 552 sites across china with largest number from Wuhan Jinyintan Hospital (132)  **Location(s):** Multiple cities, China  **Study period:** 11 Dec 2019 - 29 Jan 2020  **Aim:** To provide an updated analysis of clinical characteristics of Covid-19 in a cohort of patients throughout China which might help identify the defining clinical characteristics and severity of the disease.  **Study design:** Retrospective cohort study  **Sample size:** 1099  **Study COVID-19 case definition:** Severe vs non-severe. Based on American Thoracic Society and Infectious Disease Society of America guidelines for community acquired pneumonia.  **COVID-19 severity classification:** Laboratory confirmed cases (RT-PCR)  **Timing of classification of severity:** On admission  **Timing of blood sample collection:** On admission | **Number of non-severe COVID-19 cases:** 926  **Number of severe/critical COVID-19 cases:** 173  **^b^Age:** 47 (35-58) years, (mild: 45 [34-57] years, severe: 52 [40-65] years)  **Males:** 637/1096 (58.1%), (mild: 537/923[58.2%], severe: 100/173 [57.8%])  **Hypertension:** 165/1099 (15%), (mild: 124/926 [13.4%], severe/critical: 41/173 [23.7%])  **Diabetes:** 81/1099 (7.4%), (mild: 53/926 [5.7%], severe/critical: 28/173 [16.2%])  **Cancer:** 10/1099 (0.9%), (mild: 7/926 [0.8%], severe/critical: 3/173 [1.7%]) | **Medium** |
| Han et al.[12] | **Hospital(s):** Renmin Hospital of Wuhan University  **Location(s):** Wuhan, China  **Study period:** 1 Feb 2020 - 18 Feb 2020  **Aim:** To analyze the clinical and laboratory parameters of severe and non-severe COVID-19 patients in order to evaluate disease severity.  **Study design:** Retrospective observational study  **Sample size:** 47  **Study COVID-19 case definition:** Laboratory confirmed cases (RT-PCR)  **COVID-19 severity classification:** Severe vs non-severe. Based on American Thoracic Society and Infectious Disease Society of America guidelines for community acquired pneumonia.  **Timing of classification of severity:** On admission  **Timing of blood sample collection:** On admission | **Number of non-severe COVID-19 cases:** 23  **Number of severe/critical COVID-19 cases:** 24  **^b^Age:** 64.91 (31-87) years, (mild: 64.74 [41-81] years, severe: 65.08 [31-87] years)  **Males:** 26/47 (55.31%), (mild: 9/23 [39.13%], severe: 17/24 [70.83%])  **Hypertension:** 18/47 (38.30%), (mild: 8/23 [34.78%], severe/critical: 10/24 [41.67%])  **Diabetes:** 7/47 (14.89%), (mild: 3/23 [13.04%], severe/critical: 4/24 [16.67%])  **Cancer:** NR | **Low** |
| Herold et al.[13] | **Hospital(s):** University Hospital, Ludwig Maximilian University of Munich  **Location(s):** Munich  **Study period:** 29 Feb 2020-27 March 2020  **Aim:** To identify variables that allow the prediction of patients with a high risk of respiratory failure and need of mechanical ventilation  **Study design:** Cross-sectional study  **Sample size:** 40  **Study COVID-19 case definition:** Laboratory confirmed cases (RT-PCR)  **COVID-19 severity classification:** Severe if PaO_2_/FiO_2_ <150mmHg  **Timing of classification of severity:** 13 cases requiring mechanical ventilation with median time 2 days from admission to mechanical ventilation.  **Timing of blood sample collection:** On admission | **Number of mild COVID-19 cases:** 27  **Number of severe/critical COVID-19 cases:** 13  **^b^Age:** 57 (19-81) years, (mild: 54 [19-80] years, severe/critical: 64 [45-81] years)  **Males:** 29/40 (72%), (mild: 16/27 [59%], severe/critical: 13/13 [100%])  **Hypertension:** 19/36 (53%), (mild: 10/25 [40%], severe/critical: 9/11 [82%])  **Diabetes:** 3/37 (8%), (mild: 1/25 [4%], severe/critical: 2/12 [17%])  **Cancer:** NR | **Low** |
| Hu et al.[14] | **Hospital(s):** Tianyou Hospital  **Location(s):** Wuhan  **Study period:** 8 Jan 2020 - 20 Feb 2020  **Aim:** To identify variables that allow the prediction of patients with a high risk of respiratory failure and need of mechanical ventilation  **Study design:** Retrospective cohort study  **Sample size:** 323  **Study COVID-19 case definition:** Laboratory and clinical confirmation based on criteria by WHO and CNHC. 186 (57.6%) were confirmed by RT-PCR.  **COVID-19 severity classification:** Non-severe, severe, critical based on criteria by WHO and CNHC  **Timing of classification of severity:** On admission  **Timing of blood sample collection:** On admission | **Number of mild COVID-19 cases:** 151  **Number of severe/critical COVID-19 cases:** 172  **^d^Age:** 61 (23-91) years, (mild: 56 [23-89] years, severe: 64 [23-87] years, critical: 70[44-91] years)  **Males:** 166/323 (51.4%), (mild: 75/151 [49.7%], severe/critical: 91/172 [52.9%])  **Hypertension:** 105/323 (32.5%), (mild: 39/151 [25.8%], severe/critical: 66/172 [38.4%])  **Diabetes:** 47/323 (14.6%), (mild: 14/151 [9.3%], severe/critical: 33/172 [19.2%])  **Cancer:** 5/323 (1.5%), (mild: 0/151 [0%], severe/critical: 5/172 [2.9%]) | **Medium** |
| Lescure et al.[15] | **Hospital(s):** Bichat-Claude Bernard University Hospital (Paris, France) and Pellegrin University Hospital (Bordeaux, France)  **Location(s):** Paris and Bordeaux  **Study period:** 23 Jan 2020 - 14 Feb 2020  **Aim:** To report the relevant features of the first cases in Europe of confirmed infection, named COVID-19, with the first patient diagnosed with the disease on Jan 24, 2020.  **Study design:** Case series  **Sample size:** 5  **Study COVID-19 case definition** Laboratory confirmed cases (RT-PCR)  **COVID-19 severity classification:** Mild, severe, critical based on WHO guidelines  **Timing of classification of severity:** On admission and in the wards  **Timing of blood sample collection:** Earliest lab value after admission if mild, closest lab value following diagnosis if severe/critical. | **Number of mild COVID-19 cases:** 2  **Number of severe/critical COVID-19 cases:** 3  **^b^Age:** 46 (30-80) years, (mild: 38 [30-46] years, severe/critical: 48 [31-80] years)  **Males:** 3/5 (60.0%), (mild: 0/2 [0%], severe/critical: 3/3 [100%])  **Hypertension:** 1/5 (20%), (mild: 0 [0%], severe/critical: 1/3 [33.33%])  **Diabetes:** 0 (0%)  **Cancer:** 1/5 (20%), (mild: 0 [0%]), severe/critical: 1/3 [33.33%)] | **Low** |
| Liu Chuan et al.[16] | **Hospital(s):** 7 new coronavirus infection designated admission hospitals (Lanzhou University First Hospital, Shenyang Sixth People's Hospital, Ankang Central Hospital, Lishui Central Hospital, Zhenjiang Third People's Hospital, Baoding People's Hospital, Linxiazhou People's Hospital)  **Location(s):** Lanzhou, Shanghai, Ankang, Lishui, Zhenjiang, Baoding and Linxia.  **Study period:** 23 Jan 2020 – 8 Feb 2020  **Aim:** To retrospectively analyze the clinical features and liver function of new coronavirus pneumonia cases, and to initially explore the relationship between different clinical types of new coronavirus pneumonia and liver function damage.  **Study design:** Retrospective cohort study  **Sample size:** 32  **Study COVID-19 case definition:** Laboratory confirmed cases (nucleic acid test)  **COVID-19 severity classification:** Mild, severe and critical based on criteria by CNHC  **Timing of classification of severity:** NR  **Timing of blood sample collection:** NR | **Number of non-severe COVID-19 cases:** 28  **Number of severe/critical COVID-19 cases:** 4  **^b^Age:** 38.50 (26.25-45.75) years  **Males:** 20/32 (62.5%)  **Hypertension:** 1/32 (3.1%)  **Diabetes:** NR  **Cancer:** 2/32 (6.25%) | **High** |
| Liu Jingyuan et al.[17] | **Hospital(s):** Beijing Ditan Hospital  **Location(s):** Beijing, China  **Study period:** 13 Jan 2020 - 31 Jan 2020  **Aim:** The study aimed to select the most useful prognostic factor for severe illness incidence.  **Study design:** Prospective cohort study  **Sample size:** 61  **Study COVID-19 case definition:** Laboratory confirmed cases (RT-PCR)  **COVID-19 severity classification:** Mild, moderate, severe, critical based on criteria by CNHC  **Timing of classification of severity:** On admission  **Timing of blood sample collection:** On admission | **Number of non-severe COVID-19 cases:** 44  **Number of severe/critical COVID-19 cases:** 17  **^d^Age:** [mild: 41.0 (1.0-76.0) years, severe: 56.0 (34.0-73.0) years]  **Males:** 31/61 (50.8%), (mild: 21/44 [47.7%], severe: 10/17 [58.8%])  **Hypertension:** 12/61 (19.7%), (mild: 6/44 [13.6%], severe/critical: 6/17 [35.3%])  **Diabetes:** 5/61 (8.2%), (mild: 2/44 [4.5%], severe/critical: 3/17 [17.6%])  **Cancer:** NR | **Low** |
| Liu Min et al.[18] | **Hospital(s):** Affiliated hospital of Jianghan University  **Location(s):** Wuhan, China  **Study period:** Jan 2020  **Aim:** To explore the clinical features of new coronavirus pneumonia (new coronary pneumonia) for medical staff.  **Study design:** Retrospective cohort study  **Sample size:** 30  **Study COVID-19 case definition:** Laboratory and clinical confirmation based on criteria by CNHC; 7 (3.33%) cases positive by RT-PCR.  **COVID-19 severity classification:** Mild, moderate, severe, critical based on criteria by CNHC  **Timing of classification of severity:** NR  **Timing of blood sample collection:** On admission | **Number of non-severe COVID-19 cases:** 26  **Number of severe/critical COVID-19 cases:** 4  **^c^Age:** 35.0 (8) years  **Males:** 10/30 (33.3%)  **Hypertension:** NR  **Diabetes:** NR  **Cancer:** NR | **Medium** |
| Liu Tao et al.[19] | **Hospital(s):** Union Hospital  **Location(s):** Wuhan, China  **Study period:** 21 Jan 2020 - 16 Feb 2020  **Aim:** The purpose of this study is to explore changes of markers in peripheral blood of severe COVID-19 patients, which may be of value in disease monitoring.  **Study design:** Retrospective cohort study  **Sample size:** 80  **Study COVID-19 case definition:** Laboratory confirmed cases (RT-PCR)  **COVID-19 severity classification:** Non-severe vs severe based on criteria by CNHC  **Timing of classification of severity:** On admission  **Timing of blood sample collection:** On admission | **Number of non-severe COVID-19 cases:** 11  **Number of severe/critical COVID-19 cases:** 69  **^b^Age:** 53.0 (26.0-86.0) years, (mild: 31.0 [26.0-58.0] years, severe: 56.0 [27.0-86.0] years)  **Males:** 34/80 (42.50%), (mild: 1/11[9.09%], severe: 33/69 [47.83%])  **Hypertension:** 14/80 (17.50%), (mild: 0 [0%], severe/critical: 14/69 [20.29%])  **Diabetes:** 11/80 (13.75%), (mild: 0 [0%], severe/critical: 11/69 [15.94%])  **Cancer:** 7/80 (8.75%), (mild: 3/11 [27.27%], severe/critical: 4/69 [5.80%]) | **Medium** |
| Liu Yanli et al.[20] | **Hospital(s):** Central Hospital of Wuhan  **Location(s):** Wuhan, China  **Study period:** 2 Jan 2020 – 1 Feb 2020  **Aim:** To report the clinical characteristics of COVID-19 patients with acute respiratory distress syndrome (ARDS), and further investigate the treatment and progression of ARDS in COVID-19.  **Study design:** Retrospective cohort study  **Sample size:** 109  **Study COVID-19 case definition:** Laboratory confirmed cases (RT-PCR)  **COVID-19 severity classification:** Non-severe vs severe based on Berlin definition for ARDS  **Timing of classification of severity:** NR  **Timing of blood sample collection:** On admission | **Number of non-severe COVID-19 cases:** 56  **Number of severe/critical COVID-19 cases:** 53  **^b^Age:** 55.0 (43.0-66.0) years, (mild: 49.0 [37.00-59.0] years, severe: 61.0 [52.0-70.0] years)  **Males:** 59/109 (54.1%), (mild: 31/56[55.4%], severe: 28/53 [52.8%])  **Hypertension:** 37/109 (33.9%), (mild: 16/56 [28.6%], severe/critical: 21/53 [39.6%])  **Diabetes:** 12/109 (11.0%), (mild: 1/56 [1.8%], severe/critical: 11/53 [20.8%])  **Cancer:** NR | **Medium** |
| Lo et al.[21] | **Hospital(s):** Centro Hospitalar Conde de São Januário (C.H.C.S.J.)  **Location(s):** Macau  **Study period:** 21 Jan 2020 - 16 Feb 2020  **Aim:** The purpose of this study is to evaluate the SARS-CoV-2 RNA shedding in clinical specimens and study the clinical characteristics of 10 patients with Covid-19.  **Study design:** Case series  **Sample size:** 10  **Study COVID-19 case definition:** Laboratory confirmed cases (RT-PCR)  **COVID-19 severity classification:** Mild, moderate, severe, critical based on criteria by CNHC  **Timing of classification of severity:** On admission  **Timing of blood sample collection:** On admission | **Number of non-severe COVID-19 cases:** 6  **Number of severe/critical COVID-19 cases:** 4  **^b^Age:** 54.0 (27.0-64.0) years, (mild: 37.0[19.0] years, severe: 61.0 [5.0] years)  **Males:** 3/10 (30%), (mild: 2/6[33%], severe: 1/4 [25%])  **Hypertension:** 3 (30%)  **Diabetes:** NR  **Cancer:** NR | **Low** |
| Luo et al.[22] | **Hospital(s):** Renmin Hospital of Wuhan University  **Location(s):** Wuhan  **Study period:** 30 Jan 2020 - 20 Feb 2020  **Aim:** To evaluate the potential of CRP in outcome prediction of patients with COVID-19.  **Study design:** Retrospective cohort study  **Sample size:** 298  **Study COVID-19 case definition:** NR  **COVID-19 severity classification:** Ordinary, severe, critical based on criteria by CNHC  **Timing of classification of severity:** On admission  **Timing of blood sample collection:** On admission | **Number of non-severe COVID-19 cases:** 141  **Number of severe/critical COVID-19 cases:** 157  **^b^Age:** 57.0 (40.0-69.0) years, (mild: 42.0[32-57.0] years, severe: 67.0 [57-75.0] years)  **Males:** 150/298 (50.3%), (mild: 59/141[41.8%], severe: 91/157 [58%])  **Hypertension:** 86/298 (28.9%)  **Diabetes:** 45/298 (15.1%)  **Cancer:** NR | **Low** |
| Petrilli et al.[23] | **Hospital(s):** NYU Langone Health  **Location(s):** New York, USA  **Study period:** 1 March 2020 - 2 April 2020  **Aim:** To evaluate the potential of CRP in outcome prediction of patients with COVID-19.  **Study design:** Cross sectional study  **Sample size:** 1582  **Study COVID-19 case definition:** Laboratory confirmed cases (RT-PCR)  **COVID-19 severity classification:** Non-critical vs critical illness (composite of care in the intensive care unit, use of mechanical ventilation, discharge to hospice, or death)  **Timing of classification of severity:** In the wards  **Timing of blood sample collection:** On admission | **Number of non-severe COVID-19 cases:** 932  **Number of severe/critical COVID-19 cases:** 650  **^b^Age:** [mild: 58.0(46-71.0) years, severe: 67.0 (56-77.0) years]  **Males:** [mild: 560/932 (60.1%), severe: 442/650 (68%)]  **Hypertension:** [mild: 320/932 (34.3%), severe: 257/650 (39.5%)]  **Diabetes:** [mild: 213/932 (22.9%), severe: 176/650 (27.1%)]  **Cancer:** [mild: 54/932 (5.8%), severe: 56/650 (8.6%)] | **Low** |
| Qian et al.[24] | **Hospital(s):** Five hospitals in Zhejiang province  **Location(s):** Zhejiang province  **Study period:** 20 Jan 2020 - 11 Feb 2020  **Aim:** To reveal more data on the epidemiologic and clinical characteristics of COVID-19 patients outside of Wuhan, in Zhejiang, China.  **Study design:** Retrospective case series  **Sample size:** 91  **Study COVID-19 case definition:** Laboratory and clinical confirmation based on criteria by CNHC; 3/91 clinically diagnosed  **COVID-19 severity classification:** Mild, severe, critical based on criteria by CNHC  **Timing of classification of severity:** On admission  **Timing of blood sample collection:** On admission | **Number of non-severe COVID-19 cases:** 82  **Number of severe/critical COVID-19 cases:** 9  **^b^Age:** 50.0 (36.5-57.0) years, (mild: 49.0 [35.3-56.0] years, severe: 66.0 [54.0-80.0] years)  **Males:** 37/91 (40.66%)  **Hypertension:** 15/91 (16.48%)  **Diabetes:** 8/91 (8.79%)  **Cancer:** NR | **Medium** |
| Qin et al.[25] | **Hospital(s):** Tongji Hospital  **Location(s):** Wuhan, China  **Study period:** 10 Jan 2020 - 12 Feb 2020  **Aim:** The purpose of this study is to explore changes of markers in peripheral blood of severe COVID-19 patients, which may be of value in disease monitoring.  **Study design:** Retrospective cohort study  **Sample size:** 452  **Study COVID-19 case definition:** Laboratory confirmed cases (RT-PCR)  **COVID-19 severity classification:** Mild, moderate, severe, critical based on criteria by CNHC  **Timing of classification of severity:** On admission  **Timing of blood sample collection:** On admission | **Number of non-severe COVID-19 cases:** 166  **Number of severe/critical COVID-19 cases:** 286  **^b^Age:** 58.0 (47.0-67.0) years, (mild: 53.0 [41.25-62.0] years, severe: 61.0 [51.0-69.0] years)  **Males:** 235/452 (52%0, (mild: 80/166[48.2%], severe: 155/286 [54.2%])  **Hypertension:** 135/452 (29.50%), (mild: 30/166 [18.10%], severe/critical: 105/286 [36.7%])  **Diabetes:** 75/452 (16.4%), (mild: 22/166 [13.3%], severe/critical: 53/286 [18.5%])  **Cancer:** 14/452 (3.1%), (mild: 4/166 [2.4%], severe/critical: 10/286 [3.5%]) | **Low** |
| Qu et al.[26] | **Hospital(s):** Huizhou Municipal Central Hospital  **Location(s):** Huizhou, China  **Study period:** Jan 2020 – Feb 2020  **Aim:** To study the platelet to lymphocyte ratio and its association with prognosis in patients with Covid-19.  **Study design:** Retrospective cohort study  **Sample size:** 30  **Study COVID-19 case definition:** Laboratory confirmed cases (RT-PCR)  **COVID-19 severity classification:** Mild, moderate, severe, critical based on criteria by CNHC  **Timing of classification of severity:** On admission  **Timing of blood sample collection:** On admission | **Number of non-severe COVID-19 cases:** 27  **Number of severe/critical COVID-19 cases:** 3  **^1c,b^Age:** 50.5 (36.0-65.0) years, (mild: 49.44 [14.86] years, severe: 60.0 [5.29] years)  **Males:** 16/30 (53.3%)  **Hypertension:** NR  **Diabetes:** NR  **Cancer:** NR | **Low** |
| Tabata et al.[27] | **Hospital(s):** Self-Defense Forces Central Hospital in Japan  **Location(s):** Japan  **Study period:** 11Feb 2020 -25 Feb 2020  **Aim:** To analyze the difference between non-severe and severe cases with COVID-19 as a result of the mass infection on a cruise ship “Diamond Princess” in Japan.  **Study design:** Retrospective cohort study  **Sample size:** 104  **Study COVID-19 case definition:** Laboratory confirmed cases (RT-PCR)  **COVID-19 severity classification:** Non-severe (asymptomatic and mild cases) vs severe (severe pneumonia, SpO_2_ <93%, and patients’ need for oxygen therapy).  **Timing of classification of severity:** 84 non-severe and 20 severe cases defined on admission. 8 were later classified as severe in the wards median time: 4 days; IQR, 2.5-5.  **Timing of blood sample collection:** On admission | **Number of non-severe COVID-19 cases:** 78  **Number of severe/critical COVID-19 cases:** 28  **^b^Age:** 68.0 (46.75-75.0) years, (mild: 66.5 [44.75-74.0] years, severe: 72.5 [55.25-76.5] years)  **Males:** 47/104 (45.2%), (mild: 32/78 [42.1%], severe: 15/28 [53.6%])  **Hypertension:** NR  **Diabetes:** 7/104 (7.7%)  **Cancer:** 4/104 (3.8%) | **Low** |
| Wan et al.[28] | **Hospital(s):** Chongqing University Three Gorges Hospital  **Location(s):** Chongqing  **Study period:** 23 Jan 2020 - 8 Feb 2020  **Aim:** To describe the epidemiological and clinical features, laboratory findings, radiological characteristics, treatment, and outcomes of COVID‐19 patients in northeast Chongqing.  **Study design:** Retrospective cohort study  **Sample size:** 135  **Study COVID-19 case definition:** Laboratory confirmed cases (RT-PCR)  **COVID-19 severity classification:** Mild, moderate, severe, critical based on criteria by CNHC  **Timing of classification of severity:** NR  **Timing of blood sample collection:** NR | **Number of non-severe COVID-19 cases:** 95  **Number of severe/critical COVID-19 cases:** 40  **^b^Age:** 47.0 (36.0-55.0) years, (mild: 44.0 [33.0-49.0] years, severe: 56.0 [52.0-73.0] years)  **Males:** 72/135 (53.3%), (mild: 52/95 [54.7%], severe: 21/40 [52.5%])  **Hypertension:** 13/135 (9.6%), (mild: 9/95 [9.4%], severe/critical: 4/40 [10%])  **Diabetes:** 12/135 (8.9%), (mild: 3/95 [3.1%], severe/critical: 9/40 [22.5%])  **Cancer:** 4/135 (3.0%), (mild: 1/95 [1.0%], severe/critical: 3/40 [7.5%]) | **Low** |
| Wang et al.[29] | **Hospital(s):** Union Hospital  **Location(s):** Wuhan, China  **Study period:** 16 Jan 2020 - 29 Jan 2020  **Aim:** To describe the epidemiological, clinical, laboratory, and radiologic features, treatment, and prognosis of patients with confirmed infection of SARS-CoV-2 who were hospitalized in Union Hospital, Wuhan.  **Study design:** Retrospective cohort study  **Sample size:** 69  **Study COVID-19 case definition:** Laboratory confirmed cases (RT-PCR)  **COVID-19 severity classification:** Lowest SpO2 records during admission into SpO_2_ < 90 (severe group) and SpO_2_ >= 90 (non-severe group).  **Timing of classification of severity:** The median occurrence time of lowest SpO_2_ was 1 day (IQR 0-2.0) after admission; all: 2.0(1.0-3.0), severe: 2.0(1.0-3.0), non-severe: 1.0(0-2.0).  **Timing of blood sample collection:** On admission | **Number of non-severe COVID-19 cases:** 55  **Number of severe/critical COVID-19 cases:** 14  **^b^Age:** 42.0 (35.0-62.0) years, (mild: 37.0 [32.0-51.0] years, severe: 70.5 [62.0-77.0] years)  **Males:** 32/69 (46%), (mild: 25/55[45%], severe: 7/14 [50%])  **Hypertension:** 9/69 (13.0%), (mild: 4/55 [7.0%], severe/critical: 5/14 [36%])  **Diabetes:** 7/69 (10%), (mild: 1/55 [2%], severe/critical: 6/14 [43%])  **Cancer:** 4/69 (6%), (mild: 3/55 [5%], severe/critical: 1/14 [7%]) | **Low** |
| Wu Chaomin et al.[30] | **Hospital(s):** Jinyintan Hospital  **Location(s):** Wuhan, China  **Study period:** 25 Dec 2019 - 26 Jan 2020  **Aim:** To describe the clinical characteristics and outcomes in patients with COVID-19 pneumonia who developed acute respiratory distress syndrome (ARDS) or died.  **Study design:** Retrospective cohort study  **Sample size:** 201  **Study COVID-19 case definition:** Laboratory confirmed cases (RT-PCR)  **COVID-19 severity classification:** Non ARDS vs ARDS based on WHO guidelines  **Timing of classification of severity:** All non-ARDS defined on admission, ARDS defined while in the wards median time from admission to developing ARDS was 2 days (IQR, 1-4 days).  **Timing of blood sample collection:** On admission | **Number of non-severe COVID-19 cases:** 117  **Number of severe/critical COVID-19 cases:** 84  **^b^Age:** 51.0 (43.0-60.0) years, (mild: 48.0 [40.0-54.0] years, severe: 58.5 [50.0-69.0] years)  **Males:** 128/201 (63.7%), (mild: 68/117[58.1%], severe: 60/84 [71.4%])  **Hypertension:** 39/201 (19.40%), (mild: 16/117 [13.7%], severe/critical: 23/84 [27.4%])  **Diabetes:** 22/201 (10.9%), (mild: 6/117 [5.1%], severe/critical: 16/84 [19.0%])  **Cancer:** 1/201 (0.5%) | **Low** |
| Wu Jian et al.[31] | **Hospital(s):** First People’s Hospital of Yancheng City, the Second People’s Hospital of Fuyang City, the Second People’s Hospital of Yancheng City, and the Fifth People’s Hospital of Wuxi.  **Location(s):** Yancheng, Fuyang, Wuxi, Jiangsu and Anhui province  **Study period:** 20 Jan 2020 - 19 Feb 2020  **Aim:** To assess the factors associated with the severity and prognosis of patients infected with SARS-CoV-2.  **Study design:** Retrospective cohort study  **Sample size:** 280  **Study COVID-19 case definition:** Laboratory confirmed cases (RT-PCR)  **COVID-19 severity classification:** mild group (mild, moderate), severe group (severe, critical) based on criteria by CNHC  **Timing of classification of severity:** NR  **Timing of blood sample collection:** On admission | **Number of non-severe COVID-19 cases:** 197  **Number of severe/critical COVID-19 cases:** 83  **^c^Age:** 43.12 (19.02) years, (mild: 37.55 [17.10] years, severe: 63.04 [10.20] years)  **Males:** 151/280 (53.93%), (mild: 106/197[53.81%], severe: 45/83 [54.22%])  **CVD and CeVD:** 57/280 (20.36%), (mild: 14/197 [7.11%], severe/critical: 43/83 [51.81%])  **ESD:** 34/280 (12.14%), (mild: 6/197 [3.05%], severe/critical: 28/83 [33.73%])  **Cancer:** 5/280 (1.79%), (mild: 3/197 [1.52%], severe/critical: 2/83 [2.41%]) | **Medium** |
| Xiang Jialin et al.[32] | **Hospital(s):** The First Affiliated Hospital of Zunyi Medical University and The Fourth People's Hospital of Zunyi city  **Location(s):** Zunyi, Guizhou  **Study period:** 29 Jan 2020 - 21 Feb 2020  **Aim:** To search for a potential biomarker that could rapidly and effectively identify severe cases early among Covid-19 patients.  **Study design:** Retrospective cohort study  **Sample size:** 28  **Study COVID-19 case definition:** Laboratory confirmed cases (RT-PCR)  **COVID-19 severity classification:** Mild, moderate, severe, critical based on criteria by CNHC  **Timing of classification of severity:** NR  **Timing of blood sample collection:** NR | **Number of non-severe COVID-19 cases:** 20  **Number of severe/critical COVID-19 cases:** 8  **^c^Age:** [mild: 41.0 (19) years, severe: 66.0 (22) years]  **Males:** 15/28 (53.57%), (mild: 11/20[55%], severe: 4/8 [50%])  **Hypertension:** 5/28 (17.86%), (mild: 1/20 [5.0%], severe/critical: 4/8 [50.0%])  **Diabetes:** 4/28 (14.29%), (mild: 2/20 [10.0%], severe/critical: 2/8 [25.0%])  **Cancer:** NR | **Medium** |
| Xiang Tianxin et al.[33] | **Hospital(s):** The First Affiliated Hospital of Nanchang University  **Location(s):** Jiangxi Province  **Study period:** 21 Jan 2020 - 27 Jan 2020  **Aim:** To analyze the clinical characteristics and epidemiological characteristics of patients with novel coronavirus pneumonia treated early in Jiangxi province.  **Study design:** Cross-sectional study  **Sample size:** 49  **Study COVID-19 case definition:** Laboratory confirmed cases (RT-PCR)  **COVID-19 severity classification** Mild, moderate, severe, critical based on criteria by CNHC  **Timing of classification of severity:**  On admission  **Timing of blood sample collection:** On admission | **Number of non-severe COVID-19 cases:** 40  **Number of severe/critical COVID-19 cases:** 9  **^b^Age:** 42.9 (18-78) years, (mild: 40.6 [14.3] years, severe: 53 [14.0] years)  **Males:** 33/49 [67.3%], (mild: 25/40[62.5%], severe: 8/9 [88.9%])  **Hypertension:** 6/49 (12.24%), (mild: 2/40 [5%], severe/critical: 4/9 [44.44%])  **Diabetes:** 2/49 (4.1%), (mild: 0 [0%], severe/critical: 2/9 [22.22%])  **Cancer:** NR | **Low** |
| Xu et al.[34] | **Hospital(s):** Zhongnan Hospital of Wuhan University, Chinese PLA General Hospital, Peking Union Medical College Hospital, and affiliated hospitals of Shanghai University of Medicine & Health Sciences  **Location(s):** Wuhan, Shanghai, Beijing  **Study period:** 7 Feb 2020 -28 Feb 2020  **Aim:** To describe the epidemiological and clinical characteristics of SARS-CoV-2 pneumonia without comorbidities compared to normal controls in the Chinese Han population.  **Study design:** Retrospective cohort study  **Sample size:** 69  **Study COVID-19 case definition:** Laboratory confirmed cases (RT-PCR)  **COVID-19 severity classification:** Mild, severe and critical based on WHO guidelines  **Timing of classification of severity:**  On admission  **Timing of blood sample collection:** On admission | **Number of non-severe COVID-19 cases:** 44  **Number of severe/critical COVID-19 cases:** 25  **^b^Age:** 57 (43-69) years, (mild: 49 [36-60] years, severe: 67 [58-79] years)  **Males:** 35/69 (50.7%), (mild: 22/44[50%], severe: 13/25 [52%])  **Hypertension, Diabetes, Cancer:** Patients with comorbidities are excluded | **Low** |
| Yan et al.[35] | **Hospital(s):** Hainan Hospitals  **Location(s):** Hainan, China  **Study period:** 22 Jan 2020 - 13 March 2020  **Aim:** To analyze the demographic, clinical, radiological and laboratory data of Covid-19 cases and also classify according to the severity.  **Study design:** Retrospective cohort study  **Sample size:** 168  **Study COVID-19 case definition:** Laboratory confirmed cases (RT-PCR)  **COVID-19 severity classification:** Mild, moderate, severe, critical based on criteria by CNHC  **Timing of classification of severity:** NR  **Timing of blood sample collection:** On admission | **Number of non-severe COVID-19 cases:** 132  **Number of severe/critical COVID-19 cases:** 36  **^b^Age:** 51 (36-62) years, (mild: 49 [34-60] years, severe: 61 [50.3-68] years)  **Males:** 81/168 (48.2%), (mild: 60/132[45.5%], severe: 21/36 [58.3%])  **Hypertension:** 24/168 (14.3%), (mild: 13/132 [9.8%], severe/critical: 11/36 [30.6%])  **Diabetes:** 12/168 (17.1%), (mild: 5/132 [3.8%], severe/critical: 7/36 [19.4%])  **Cancer:** 2/168 (1.2%), (mild: 2/132 [1.5%], severe/critical: 0 [0%]) | **Medium** |
| Young et al.[36] | **Hospital(s):** 4 hospitals  **Location(s):** Singapore  **Study period:** 23 Jan 2020 - 3 Feb 2020  **Aim:** To report the initial experience in Singapore with the epidemiologic investigation of this outbreak, clinical features, and management.  **Study design:** Case series  **Sample size:** 18  **Study COVID-19 case definition:** Laboratory confirmed cases (RT-PCR)  **COVID-19 severity classification:** Non-severe vs severe (requiring supplemental oxygen if SpO2 < 92%)  **Timing of classification of severity:** Day 0 for non-severe (12), median 3 days severe cases (6).  **Timing of blood sample collection:** Baseline | **Number of non-severe COVID-19 cases:** 12  **Number of severe/critical COVID-19 cases:** 6  **^d^Age:** 47 (31-73) years, (mild: 37 [31-56] years, severe: 56 [47-73] years)  **Males:** 9/18 (50%), (mild: 7/12[58.33%], severe: 2/6 [33.33%])  **Hypertension:** 4/18 (22.22%), (mild: 0 [0%], severe/critical: 4/6 [66.67%])  **Diabetes:** 1/18 (5.56%), (mild: 0 [0%], severe/critical: 1/6 [16.67%])  **Cancer:** NR | **Medium** |
| Yuan et al[37]. | **Hospital(s):** Chongqing Public Health Center for Medical Treatment  **Location(s):** Chongqing  **Study period:** 24 Jan 2020 - 23 Feb 2020  **Aim:** To retrospectively analyze the clinical characteristics, treatment options and outcomes of patients with new coronavirus pneumonia admitted in Chongqing hospital.  **Study design:** Retrospective cohort study  **Sample size:** 223  **Study COVID-19 case definition:** Laboratory confirmed cases (RT-PCR)  **COVID-19 severity classification:** Mild, moderate, severe, critical based on criteria by CNHC  **Timing of classification of severity:** On admission  **Timing of blood sample collection:** On admission | **Number of non-severe COVID-19 cases:** 192  **Number of severe/critical COVID-19 cases:** 31  **^b^Age:** 46.5 (16.1) years, (mild: 44.9 [16] years, severe: 56.14 [12.4] years)  **Males:** 105/223 (47.09%), (mild: 87/192[45.31%], severe: 18/31 [58.06%])  **Hypertension:** 25/223 (11.21%), (mild: 21/192 [10.94%], severe/critical: 4/31 [12.90%])  **Diabetes:** 18/223 (8.07%), (mild: 10/192 [5.21%], severe/critical: 8/31 [25.81%])  **Cancer:** NR | **Low** |
| Zeng et al.[38] | **Hospital(s):** Shenzhen Third People’s hospital  **Location(s):** Shenzhen  **Study period:** 11 Jan 2020 - 28 Feb 2020  **Aim:** To compare the characteristics between patients with severe and non-severe outcome and analyze to assess the risk of progression to severe conditions.  **Study design:** Retrospective cohort study  **Sample size:** 338  **Study COVID-19 case definition:** Laboratory confirmed cases (RT-PCR)  **COVID-19 severity classification:** Mild, moderate, severe, critical based on criteria by CNHC  **Timing of classification of severity:** On admission for non-severe, at mean 3.7 days (3.0) post admission for severe cases  **Timing of blood sample collection:** within 1 day of admission | **Number of non-severe COVID-19 cases:** 262  **Number of severe/critical COVID-19 cases:** 76  **^c^Age:** 49.0 (14.5) years, (mild: 46.1 [14.1] years, severe: 58.7 [11.0] years)  **Males:** 162/338 (47.9%), (mild: 115/262[43.9%], severe: 47/76 [61.8%])  **Hypertension:** 51/338 (15.1%), (mild: 30/262 [11.5%], severe/critical: 21/76 [27.6%])  **Diabetes:** 25/338 (7.4%), (mild: 12/262 [4.6%], severe/critical: 13/76 [17.1%])  **Cancer:** 2/338 (0.6%), (mild: 1/262 [0.4%], severe/critical: 1/76 [1.3%]) | **Low** |
| Zhang Gemin et al.[39] | **Hospital(s):** Xinzhou District People’s Hospital  **Location(s):** Wuhan Xinzhou District  **Study period:** 16 Jan 2020 - 25 Feb 2020  **Aim:** To analyze the clinical characteristics and laboratory findings of 95 cases with 2019 novel coronavirus pneumonia excluding patients with severe underlying diseases.  **Study design:** Retrospective cohort study  **Sample size:** 95  **Study COVID-19 case definition:** Laboratory confirmed cases (RT-PCR)  **COVID-19 severity classification:** Mild, moderate, severe, critical based on criteria by CNHC  **Timing of classification of severity:** NR  **Timing of blood sample collection: H**ighest or lowest value (where appropriate) while in the wards | **Number of non-severe COVID-19 cases:** 63  **Number of severe/critical COVID-19 cases:** 32  **^b^Age:** 49.0 (39.0-58.0) years, (mild: 49 [41-57] years, severe: 50.5 [38.3-58.8] years)  **Males:** 53/95 (55.8%), (mild: 32/63[50.8%], severe: 21/32 [65.6%])  **Hypertension:** NR  **Diabetes:** NR  **Cancer:** NR | **Medium** |
| Zhang Guqin et al.[40] | **Hospital(s):** Zhongnan Hospital of Wuhan University  **Location(s):** Wuhan  **Study period:** 2 Jan 2020 - 10 Feb 2020  **Aim:** To study the epidemiology, clinical features and outcomes of patients with COVID-19.  **Study design:** Retrospective cohort study  **Sample size:** 221  **Study COVID-19 case definition:** Laboratory confirmed cases (RT-PCR)  **COVID-19 severity classification:** Mild, moderate, severe, critical based on criteria by CNHC  **Timing of classification of severity:** On admission  **Timing of blood sample collection:** On admission | **Number of non-severe COVID-19 cases:** 166  **Number of severe/critical COVID-19 cases:** 55  **^d^Age:** 55.0 (39.0-66.5) years, (mild: 51.0 [36.0-64.3] years, severe: 62.0 [52.0-74.0] years)  **Males:** 108/221 (48.9%), (mild: 73/166[44%], severe: 35/55 [63.6%])  **Hypertension:** 54/221 (24.4%), (mild: 28/166 [16.9%], severe/critical: 26/55 [47.3%])  **Diabetes:** 22/221 (10%), (mild: 15/166 [9.0%], severe/critical: 7/55 [12.7%])  **Cancer:** 9/221 (4.1%), (mild: 5/166 [3.0%], severe/critical: 4/55 [7.3%]) | **Low** |
| Zhang Huizheng et al.[41] | **Hospital(s):** Chongqing Public Health Medical Center  **Location(s):** Chongqing  **Study period:** 11 Feb 2020 - 28 Feb 2020  **Aim:** To study the effective indicators for predicting disease severity in SARS-CoV-2 infected patients.  **Study design:** Retrospective cohort study  **Sample size:** 43  **Study COVID-19 case definition:** Laboratory confirmed cases (RT-PCR)  **COVID-19 severity classification:** Mild, moderate, severe, critical based on criteria by CNHC  **Timing of classification of severity:** On admission  **Timing of blood sample collection:** On admission | **Number of non-severe COVID-19 cases:** 29  **Number of severe/critical COVID-19 cases:** 14  **^c^Age:** [mild: 44.34 (15.84) years, severe: 61.70 (9.22) years]  **Males:** 22/43 (51.2%), (mild: 17/29 [58.6%], severe: 5/14 [35.7%])  **Hypertension:** 4/43 (9.3%), (mild: 3/29 [10.3%], severe/critical: 1/14 [7.1%])  **Diabetes:** 6/43 (14%), (mild: 3/29 [10.3%], severe/critical: 3/14 [21.4%])  **Cancer:** NR | **Medium** |
| Zhang Jin-jin et al.[42] | **Hospital(s):** No. 7 Hospital of Wuhan  **Location(s):** Wuhan  **Study period:** 15 Jan 2020 - 3 Feb 2020  **Aim:** To investigate the clinical characteristic and allergy status of patients infected with SARS-CoV-2.  **Study design:** Retrospective cohort study  **Sample size:** 140  **Study COVID-19 case definition:** Laboratory confirmed cases (RT-PCR)  **COVID-19 severity classification:** Mild, moderate, severe, critical based on criteria by CNHC  **Timing of classification of severity:** On admission  **Timing of blood sample collection:** On admission | **Number of non-severe COVID-19 cases:** 82  **Number of severe/critical COVID-19 cases:** 58  **^d^Age:** 57.0 (25.0-87) years, (mild: 51.5 [26-78] years, severe: 64.0 [25-87] years)  **Males:** 71/140 (50.7%), (mild: 38/82 [46.3%], severe: 33/58 [56.9%])  **Hypertension:** 42/140 (30%), (mild: 20/82 [24.4%], severe/critical: 22/58 [37.9%])  **Diabetes:** 17/140 (12.1%), (mild: 9/82 [11.0%], severe/critical: 8/58 [13.8%])  **Cancer:** NR | **Medium** |
| Zhao et al.[43] | **Hospital(s):** Beijing YouAn Hospital  **Location(s):** Beijing  **Study period:** 21 Jan 2020 - 8 Feb 2020  **Aim:** To give the information on clinical characteristics and different durations of COVID-19 and to identify the potential risk factors for longer hospitalization duration.  **Study design:** Retrospective cohort study  **Sample size:** 77  **Study COVID-19 case definition:** Laboratory confirmed cases (RT-PCR)  **COVID-19 severity classification:** Non-severe vs severe based on criteria by WHO  **Timing of classification of severity:** On admission  **Timing of blood sample collection:** On admission | **Number of non-severe COVID-19 cases:** 57  **Number of severe/critical COVID-19 cases:** 20  **^c^Age:** 52 (20) years, (mild: 45 [17] years, severe: 69 [15] years)  **Males:** 34/77 (44.2%), (mild: 23/57[40.4%], severe: 11/20 [55.0%])  **Hypertension:** 16/77 (20.8%), (mild: 8/57 [14%], severe/critical: 8/20 [40.0%])  **Diabetes:** 6/77 (7.8%), (mild: 4/57 [7.0%], severe/critical: 2/20 [10.0%])  **Cancer:** 4/77 (5.2%), (mild: 3/57 [5.3%], severe/critical: 1/20 [5.0%]) | **Low** |
| Zheng et al.[44] | **Hospital(s):** The First Affiliated Hospital of Anhui Medical University and Fuyang second people's Hospital  **Location(s):** Hefei, Fuyang  **Study period:** NR  **Aim:** To study the functional exhaustion of antiviral lymphocytes in Covid-19 patients.  **Study design:** Retrospective cohort study  **Sample size:** 68  **Study COVID-19 case definition:** Laboratory confirmed cases (RT-PCR)  **COVID-19 severity classification:** Mild vs severe, criteria NR  **Timing of classification of severity:** NR  **Timing of blood sample collection:** On admission | **Number of non-severe COVID-19 cases:** 55  **Number of severe/critical COVID-19 cases:** 13  **^d^Age:** 47.13 (11-84) years, (mild: 43.93 [11-84] years, severe: 60.69 [59-82] years)  **Males:** 36/68 (52.9%), (mild: 26/55[47.2%], severe: 10/13 [76.9%])  **Hypertension:** NR  **Diabetes:** NR  **Cancer:** NR | **Medium** |
| Zhou et al.[45] | **Hospital(s):** Central Hospital Wuhan  **Location(s):** Wuhan  **Study period:** 1 Jan 2020 - 28 Feb 2020  **Aim:** To elucidate a novel predictor of disease severity in patients with coronavirus disease-19 (COVID-19) infected by SARS-CoV-2.  **Study design:** Cross sectional Study  **Sample size:** 377  **Study COVID-19 case definition:** Laboratory confirmed cases (RT-PCR)  **COVID-19 severity classification:** Severe pneumonia vs non-severe pneumonia.  **Timing of classification of severity:** On admission  **Timing of blood sample collection:** Within one day of admission | **Number of non-severe COVID-19 cases:** 260  **Number of severe/critical COVID-19 cases:** 117  **^c^Age:** [mild: 48.35 (16.17) years, severe: 65.63 (14.03) years]  **Males:** [mild: 102/260(39.23%), severe: 68/117 (58.12%)]  **Hypertension:** [mild: 63/260 (24.23%), severe: 70/117 (59.83%)]  **Diabetes:** [mild: 42/260 (16.15%), severe: 42/117 (35.9%)]  **Cancer:** NR | **Low** |

ARDS: Acute Respiratory Distress Syndrome

CVD and CeVD: Cardiovascular Disease and Cerebrovascular Disease

CNHC: China National Health Commission

WHO: World Health Organisation

ESD: Endocrine System Disease

NR: Not Reported

Age is reported as ^c^mean (SD) / ^b^median (IQR) / ^d^median (range)

^a^Overall Risk of Bias by professional judgement and consensus by authors. See Supplementary File S4 for detailed judgement

# **S4 Fig. Forest plots and Leave-one-out analysis for all laboratory parameters**


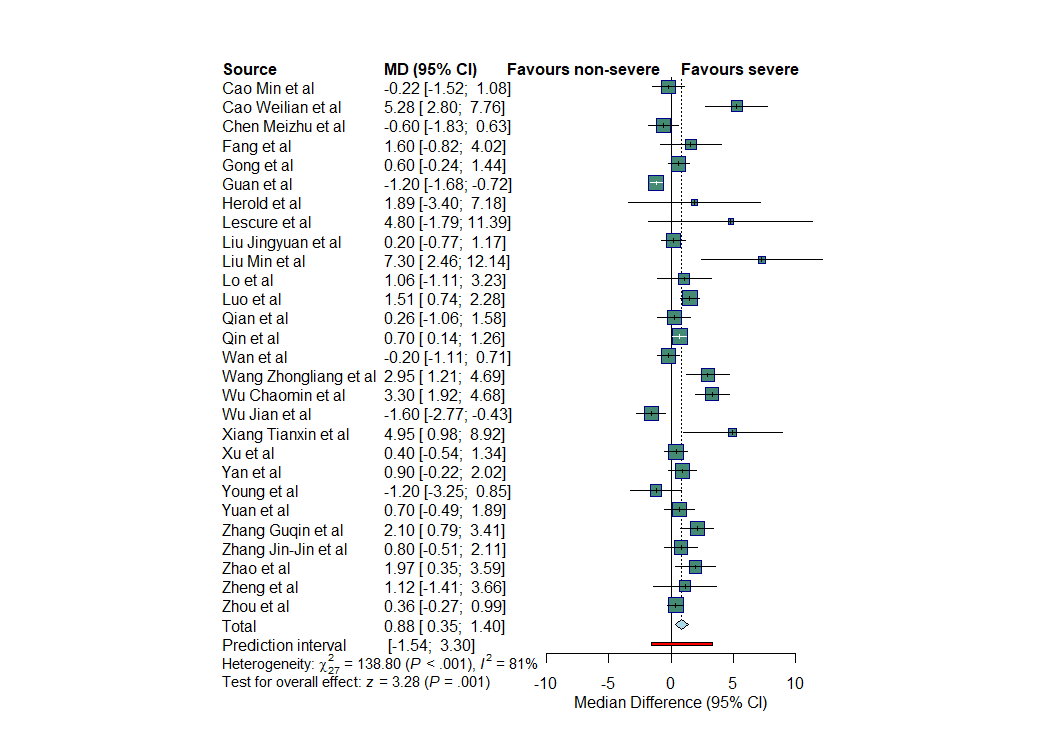

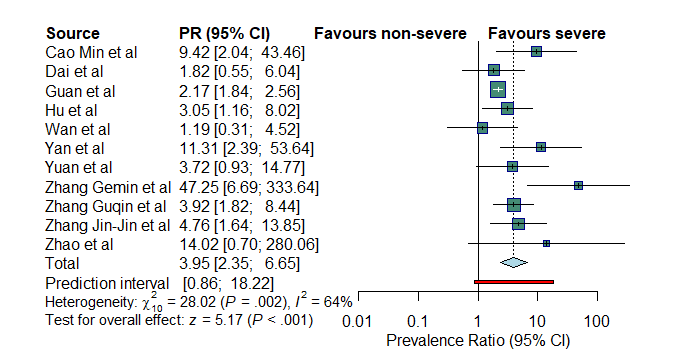

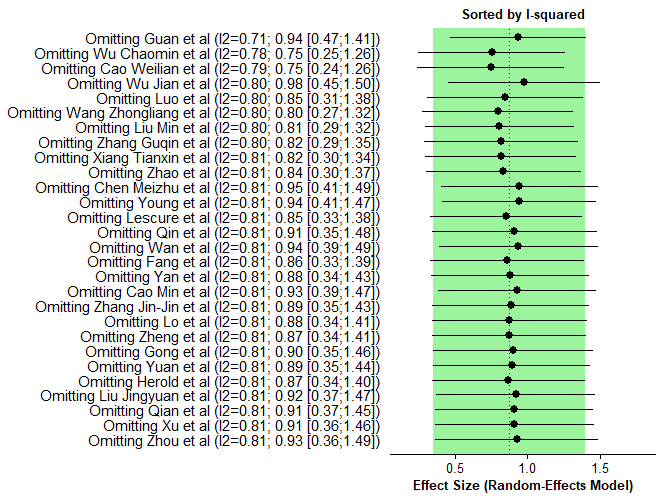

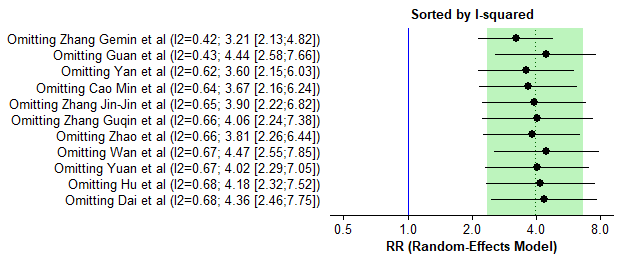


**eFig 1. Forest plots and Leave-one-out analyses for white cell count**

**eFig 2. Forest plots and Leave-one-out analyses for neutrophil count**


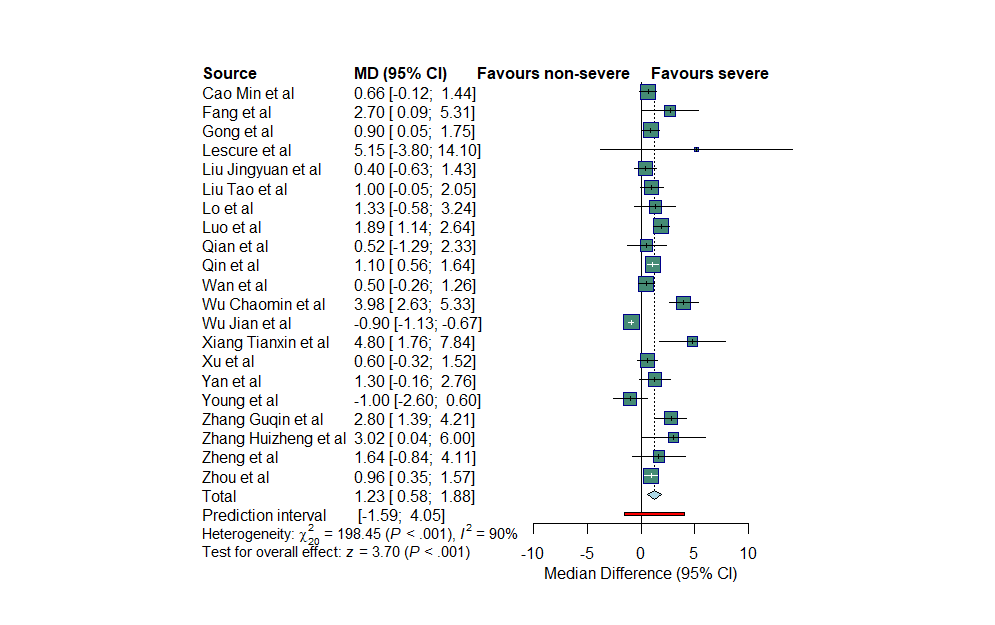

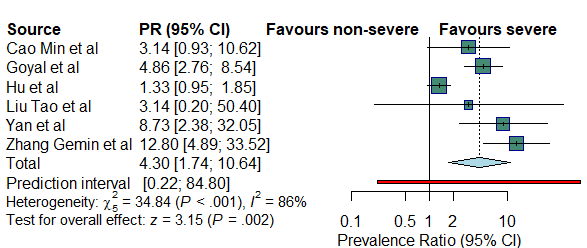

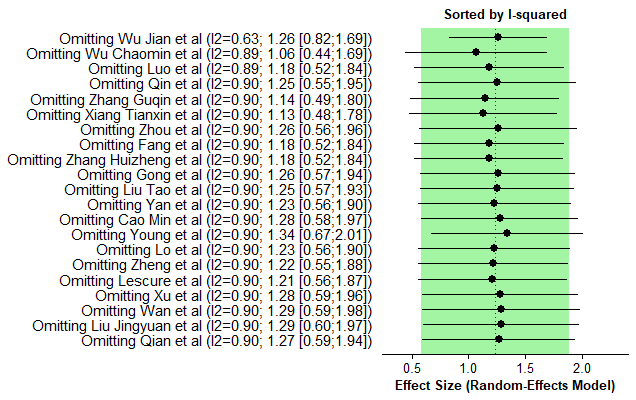

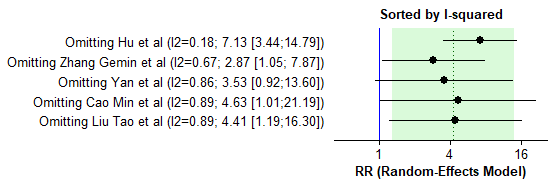

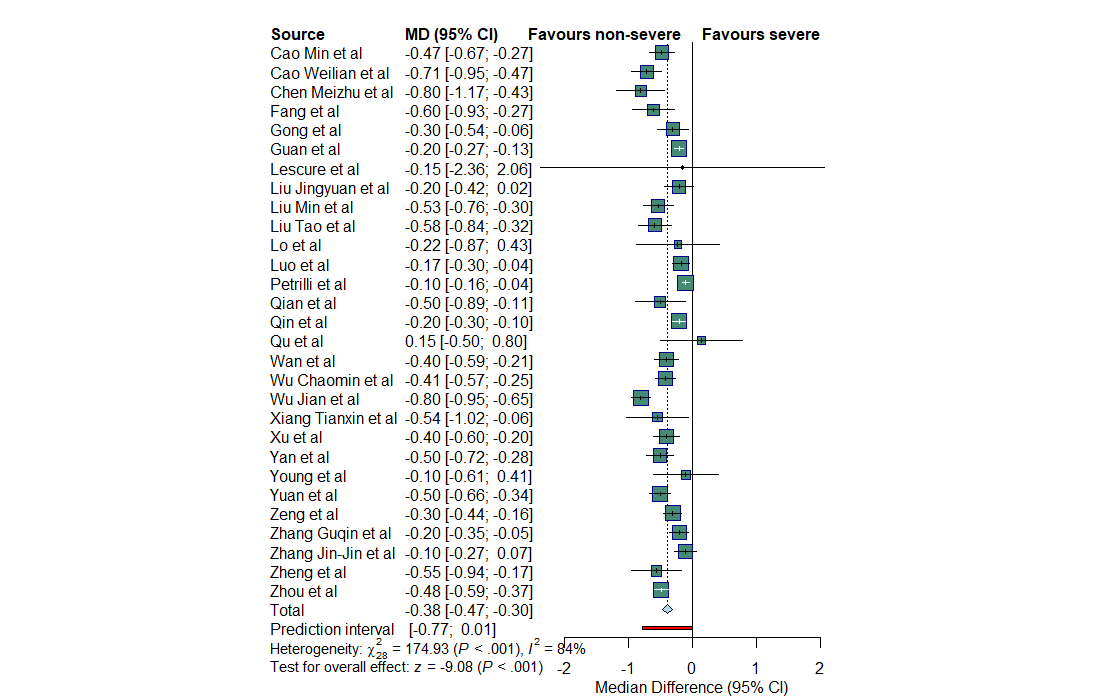

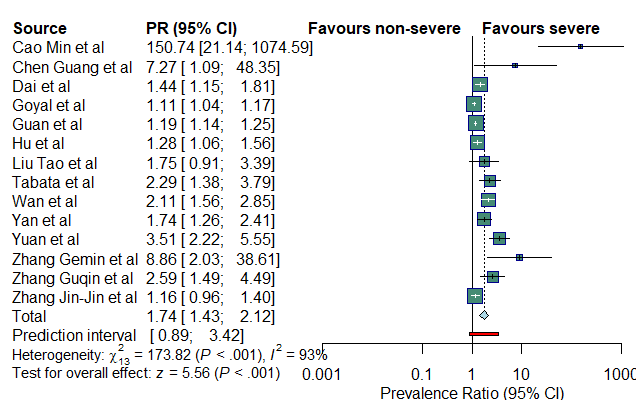

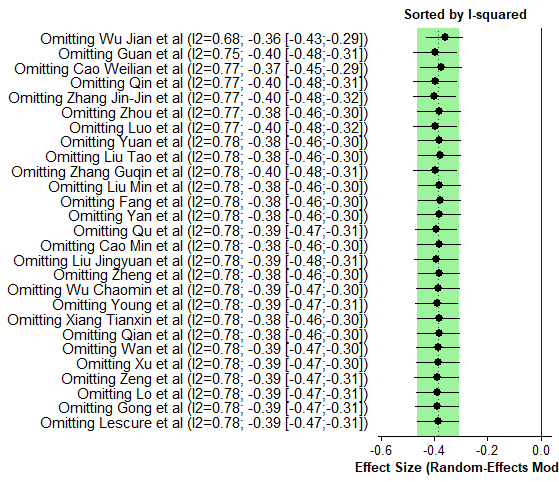

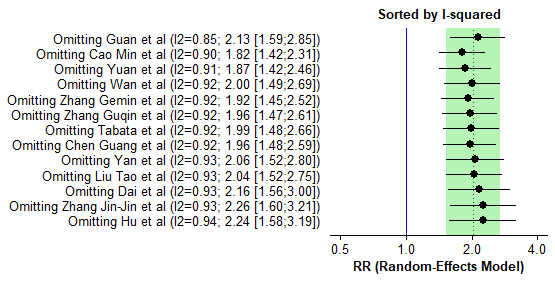


**eFig 3. Forest plots and Leave-one-out analyses for lymphocyte count**


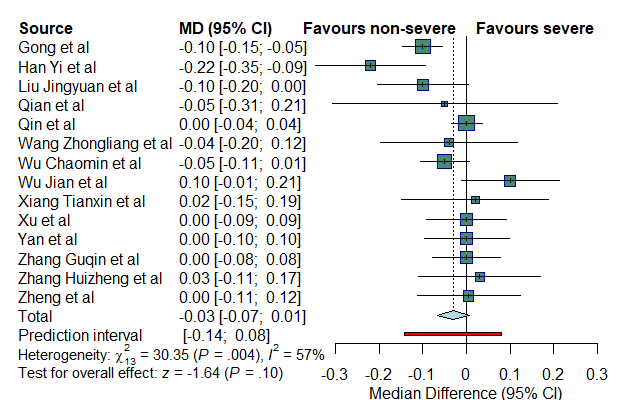

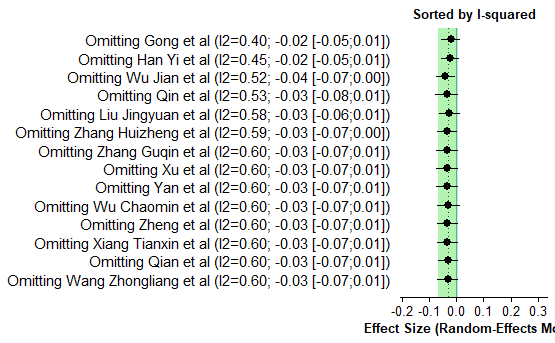


**eFig 4. Forest plot and Leave-one-out analysis for monocyte count**


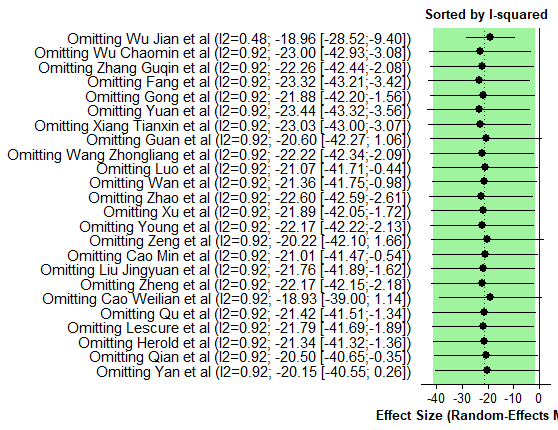

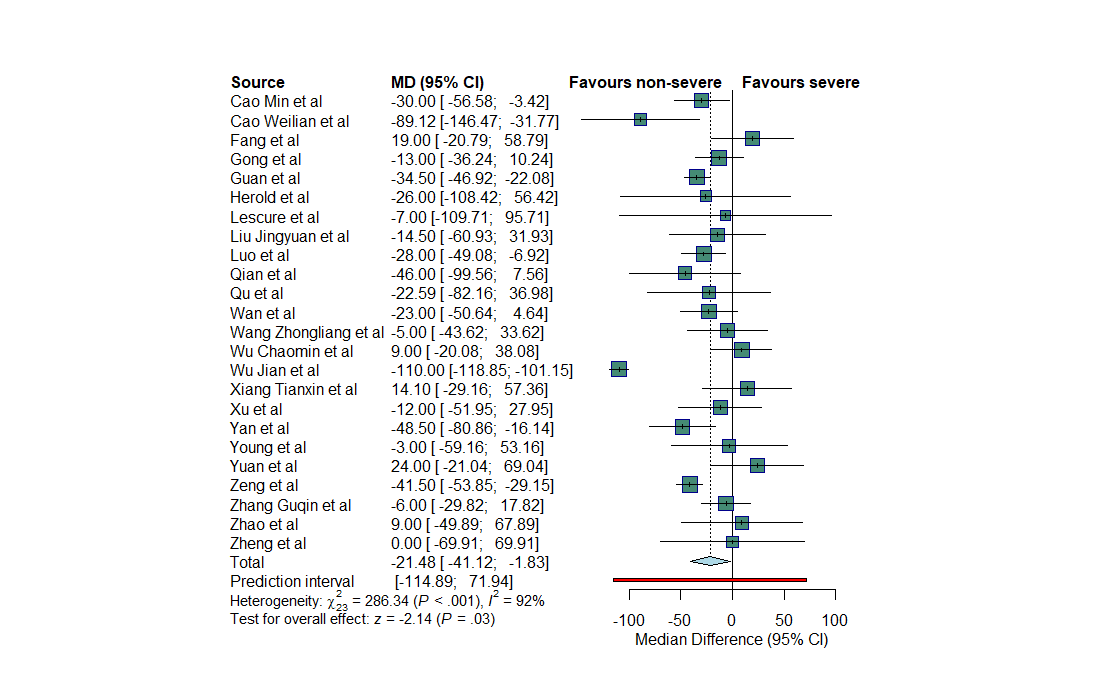

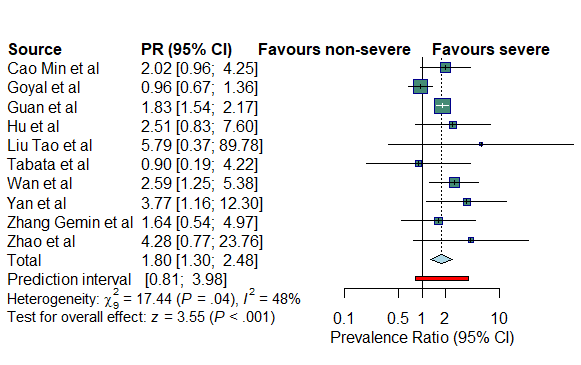

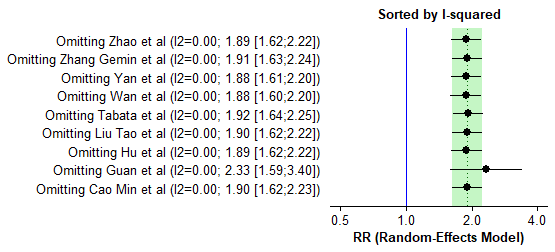


**eFig 5. Forest plots and Leave-one-out analyses for platelet count**


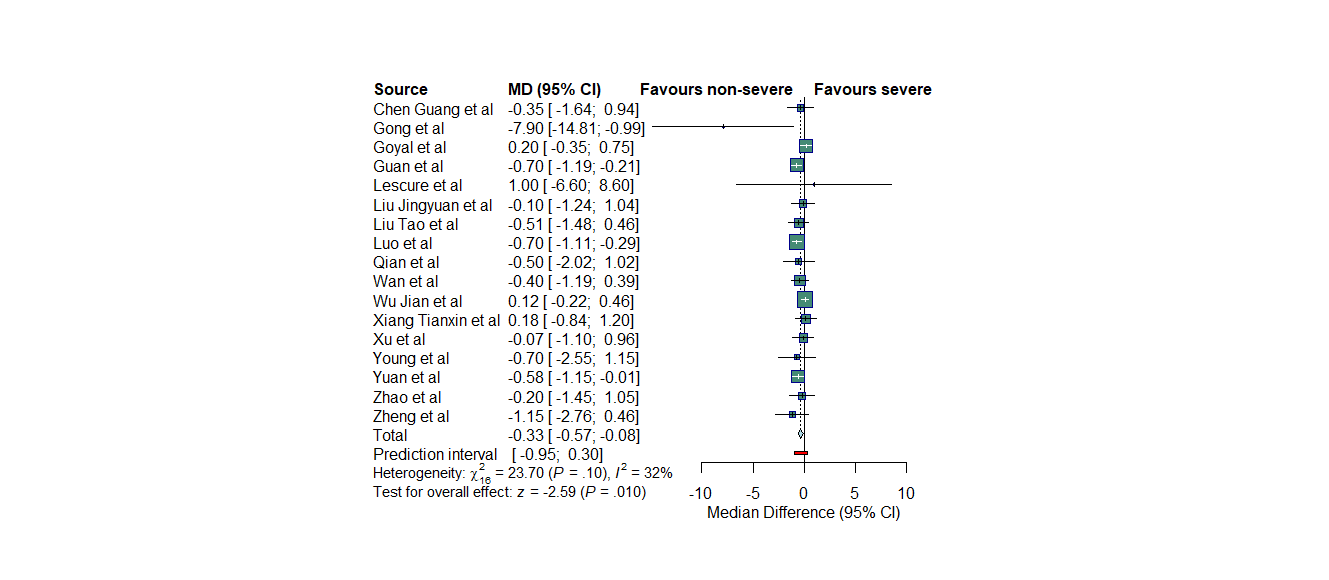

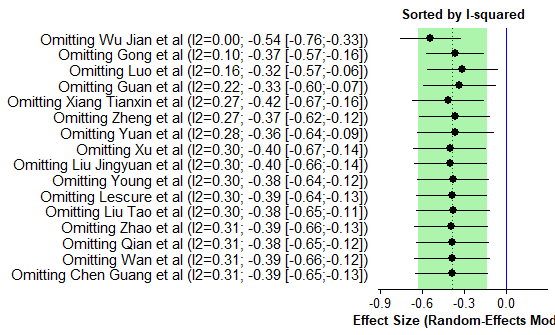


**eFig 6. Forest plot and Leave-one-out analysis for haemoglobin**


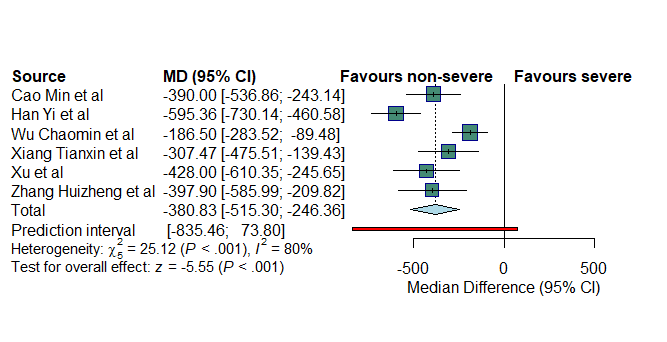

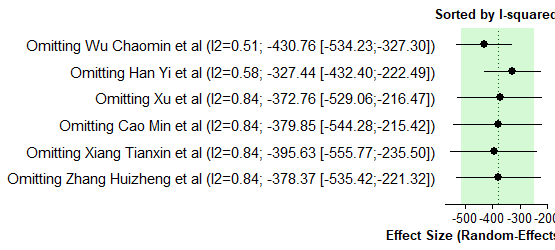


**eFig 7. Forest plot and Leave-one-out analysis for CD3**


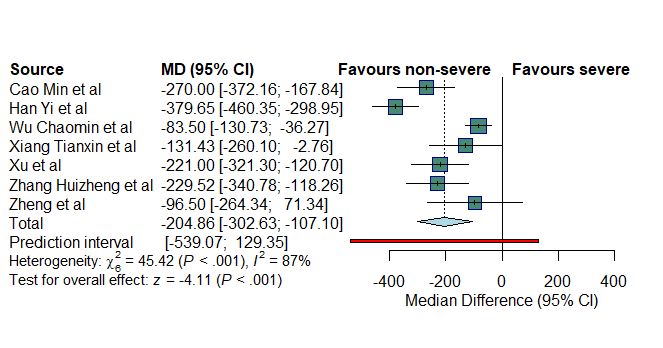

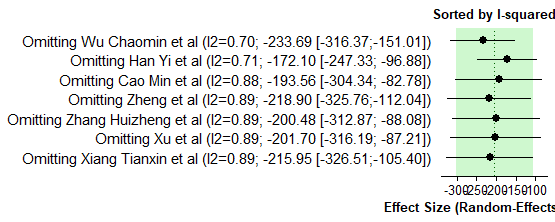


**eFig 8. Forest plot and Leave-one-out analysis for CD4**


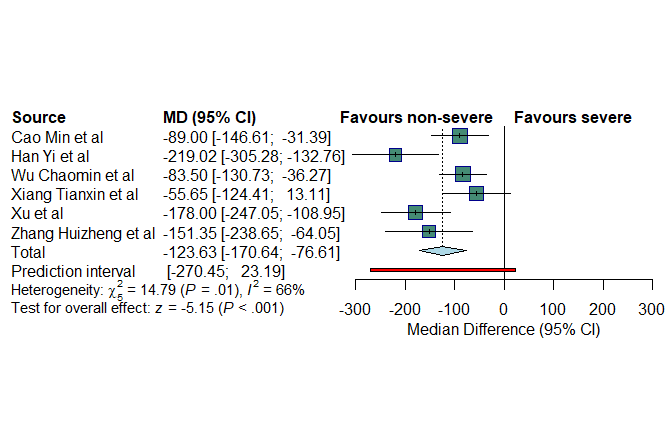

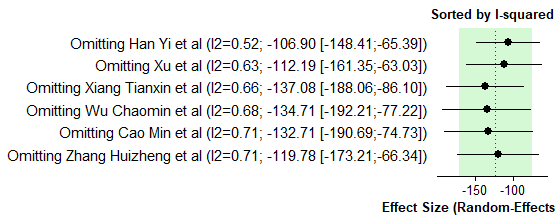


**eFig 9. Forest plot and Leave-one-out analysis for CD8**


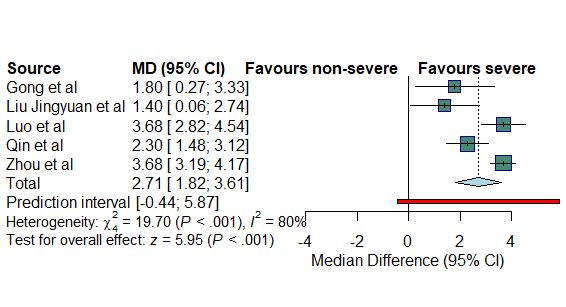

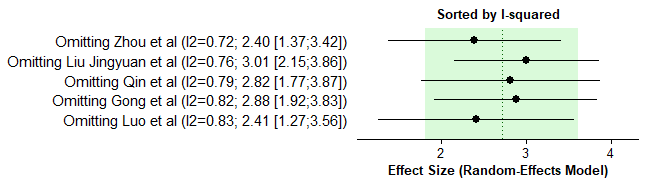


**eFig 10. Forest plot and Leave-one-out analysis for neutrophil-to-lymphocyte ratio**


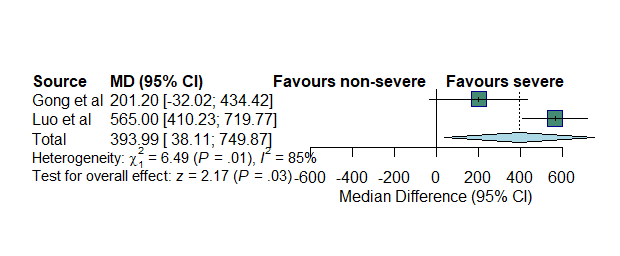

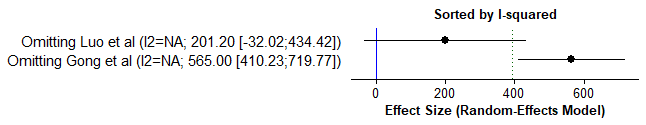


**eFig 11. Forest plot and Leave-one-out analysis for systematic inflammation index**


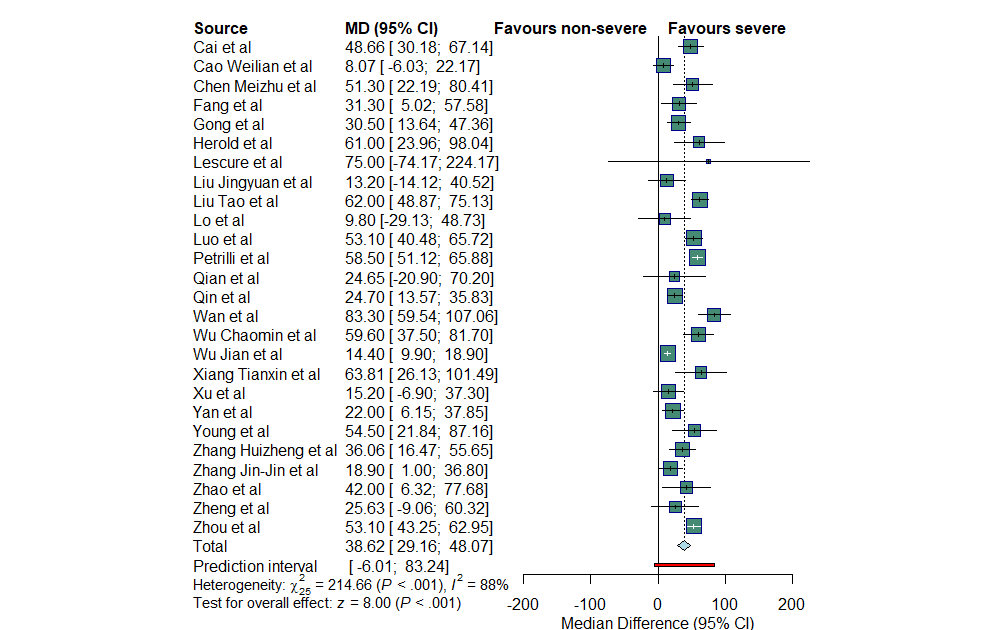

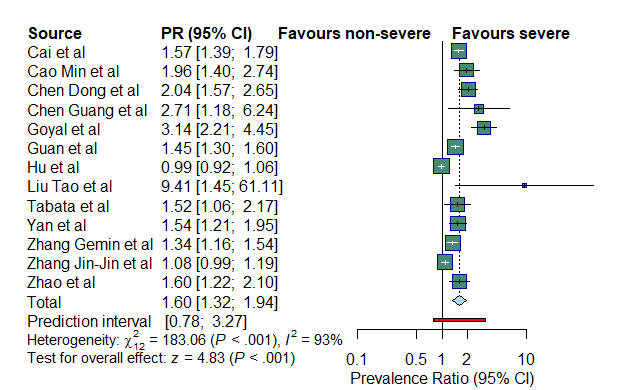

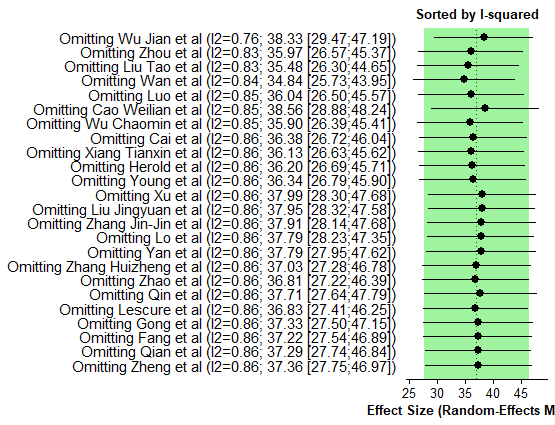

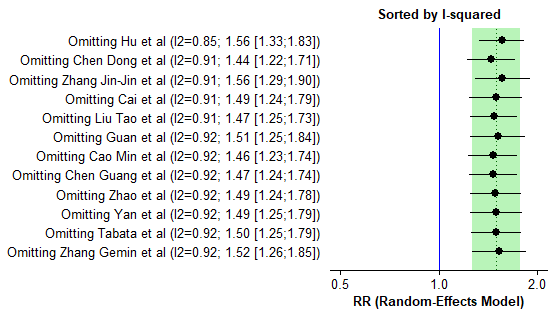


**eFig 12. Forest plots and Leave-one-out analyses for C-reactive protein**


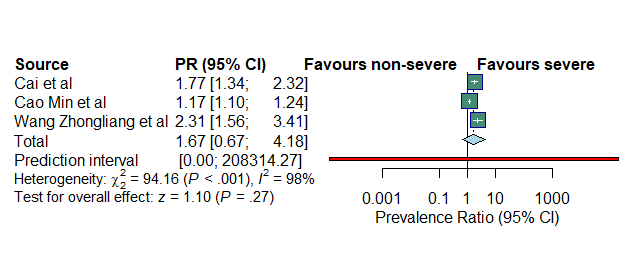

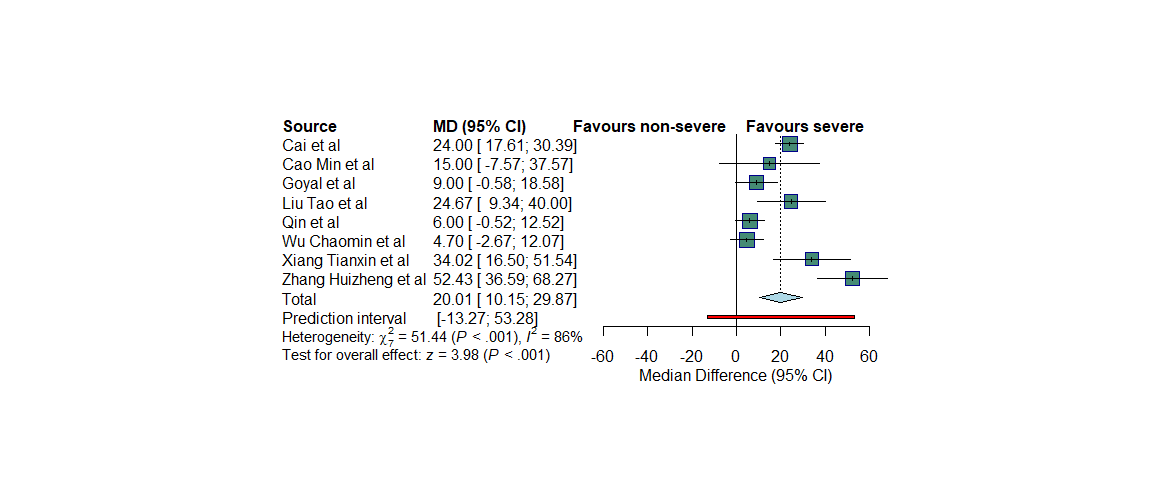

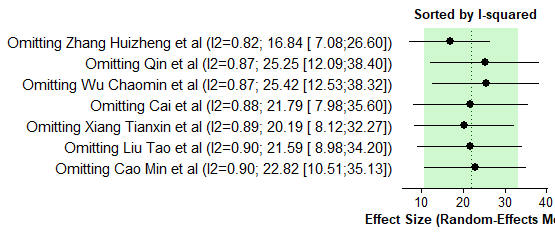

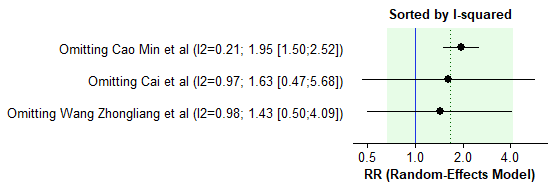


**eFig 13. Forest plots and Leave-one-out analyses for erythrocyte sedimentation rate**


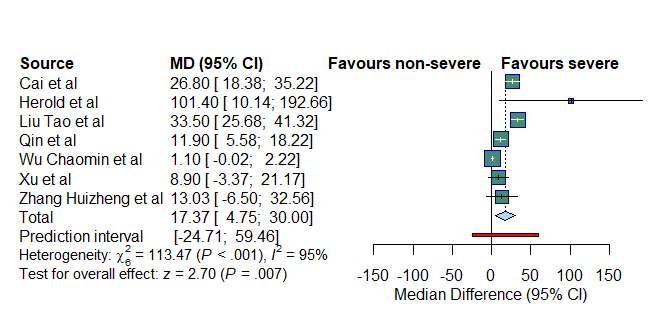

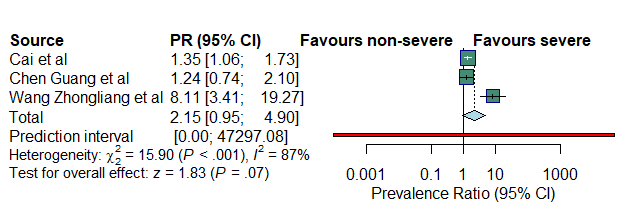

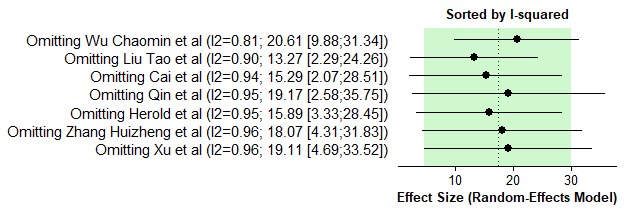

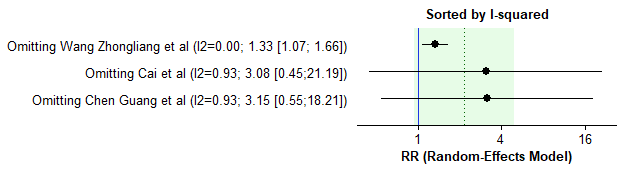


**eFig 14. Forest plots and Leave-one-out analyses for interleukin-6**


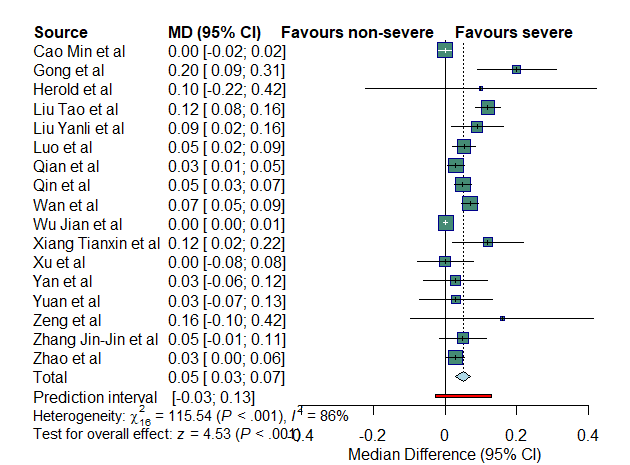

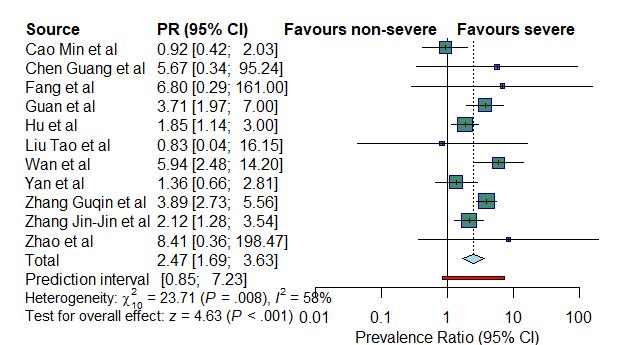

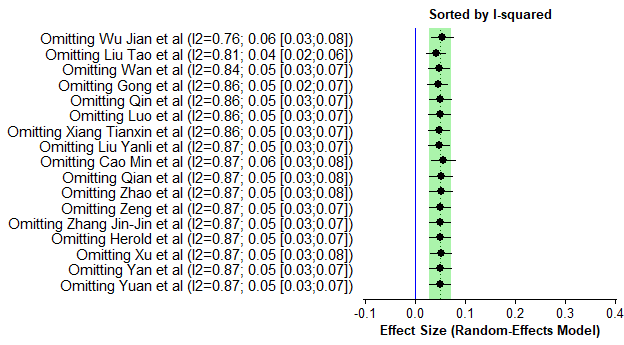

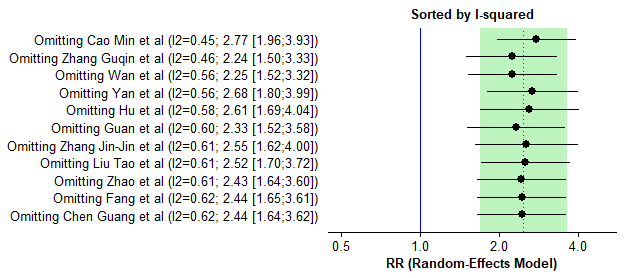


**eFig 15. Forest plots and Leave-one-out analyses for procalcitonin**


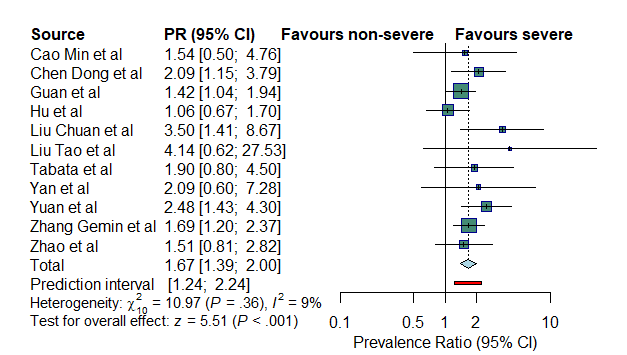

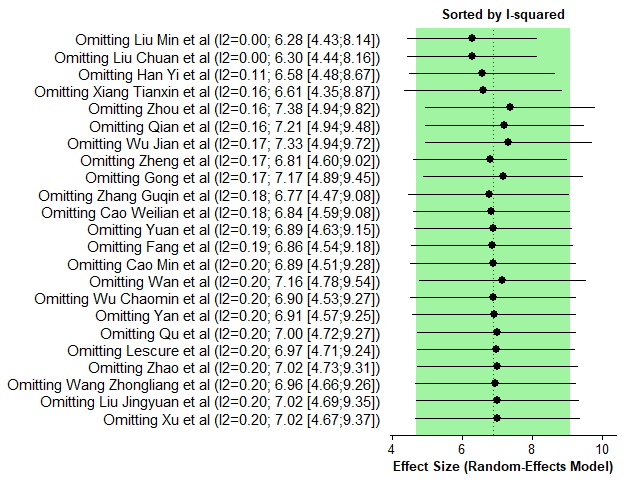

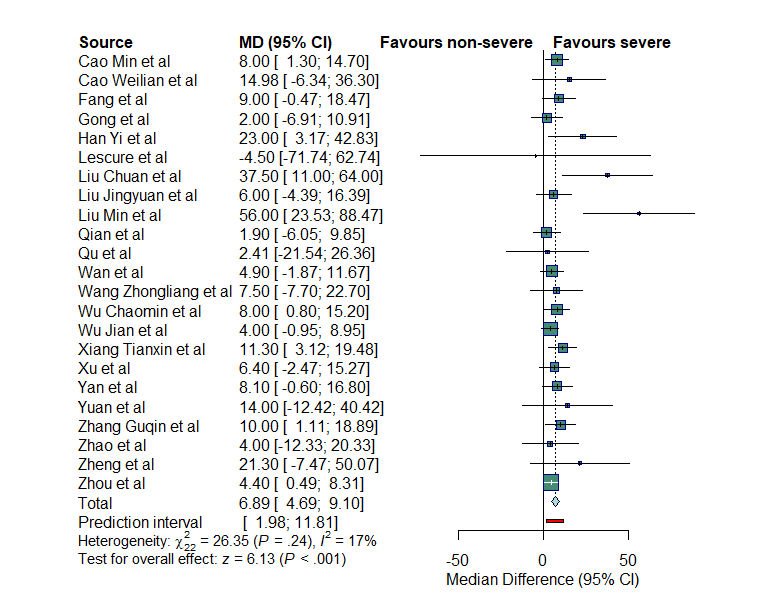

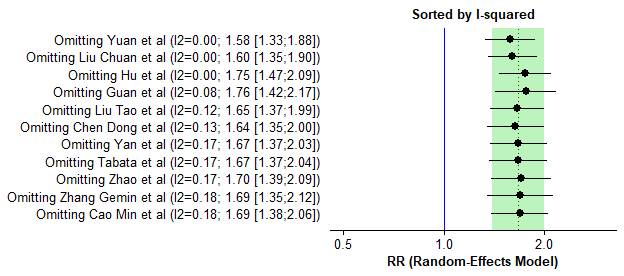


**eFig 16. Forest plots and Leave-one-out analyses for alanine aminotransferase**


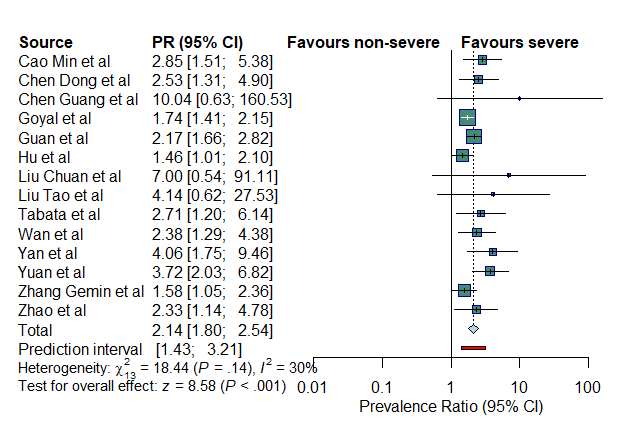

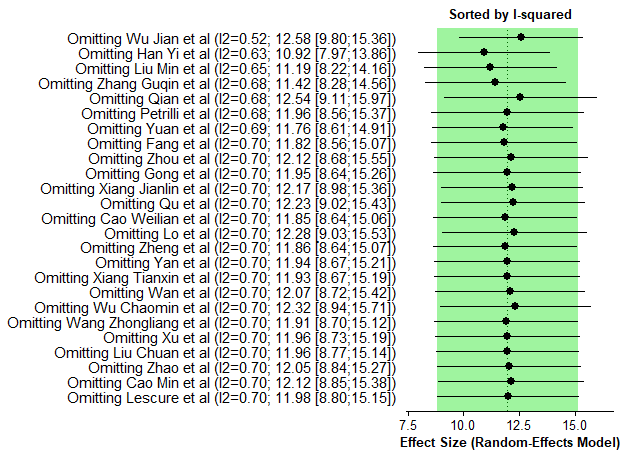

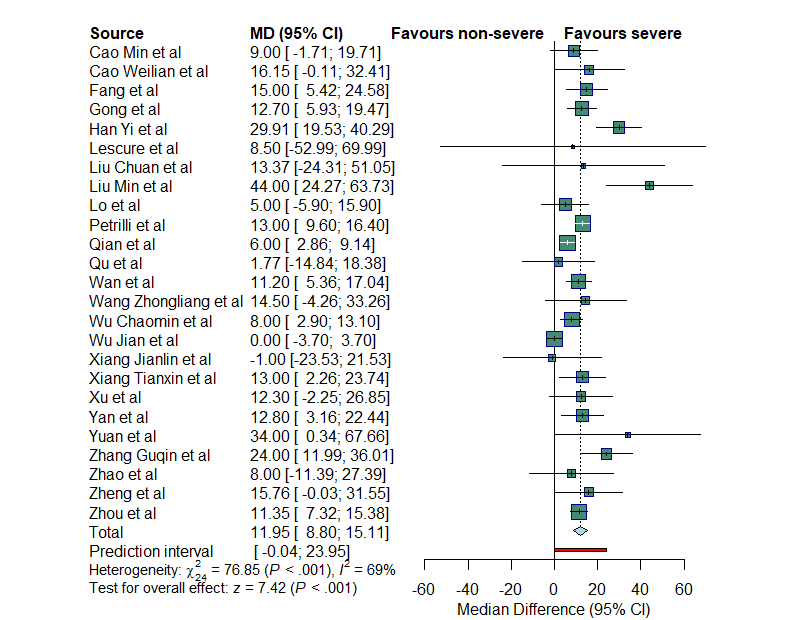

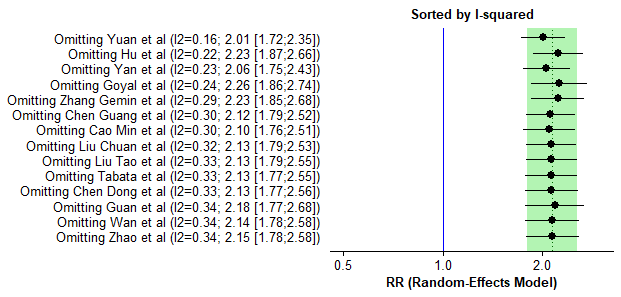


**eFig 17. Forest plots and Leave-one-out analyses for aspartate aminotransferase**


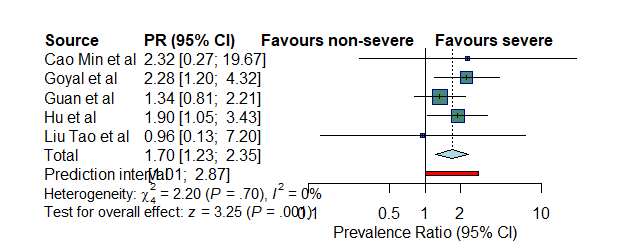

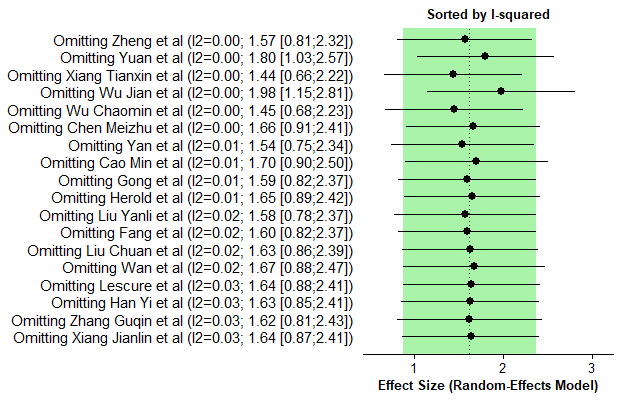

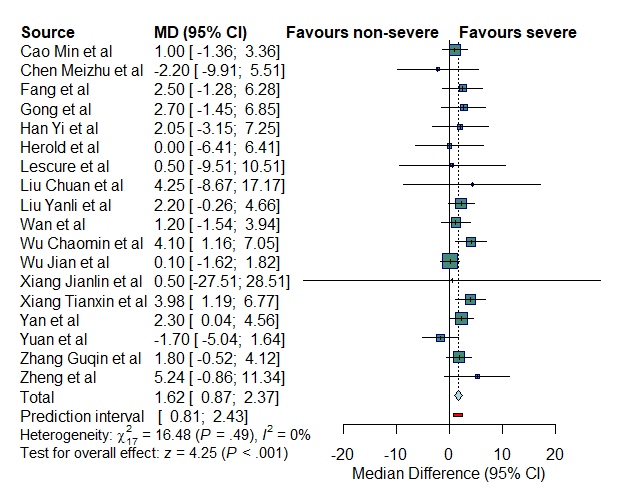

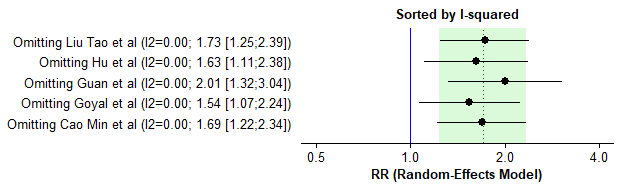


**eFig 18. Forest plots and Leave-one-out analyses for total bilirubin**


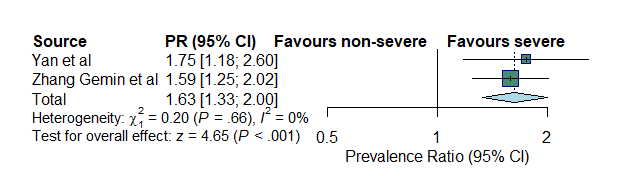

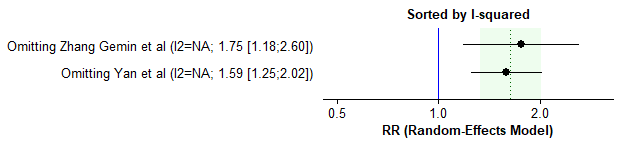

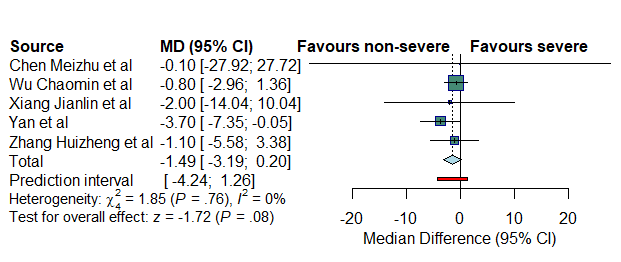

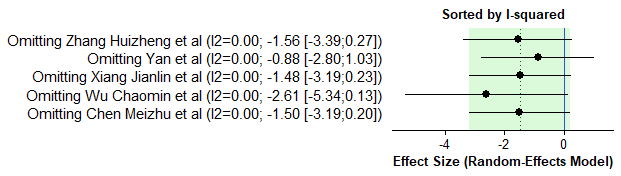


**eFig 19. Forest plots and Leave-one-out analyses for total protein**


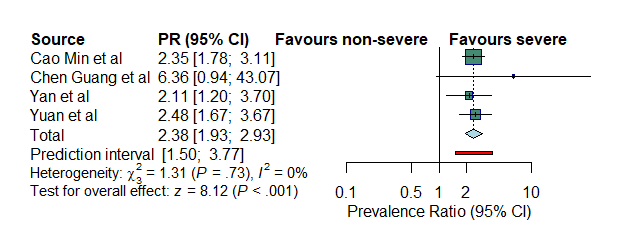

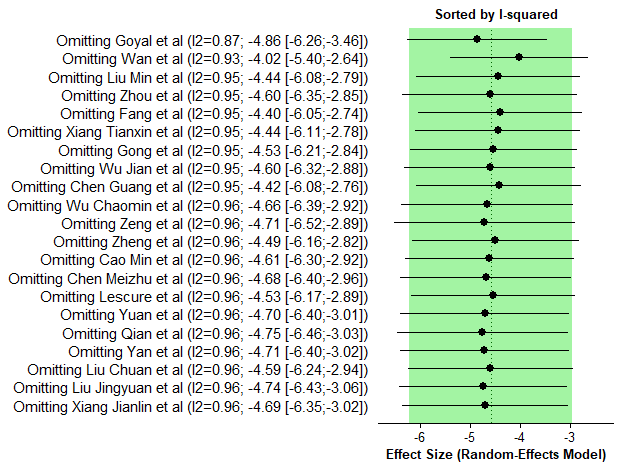

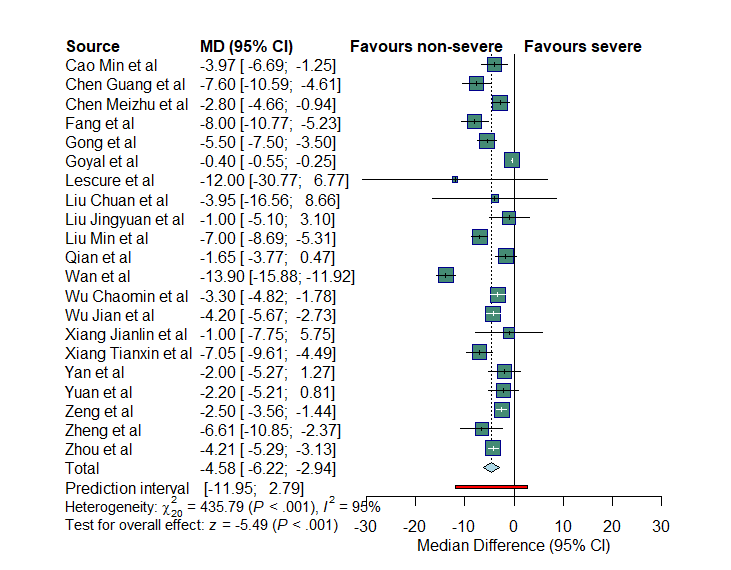

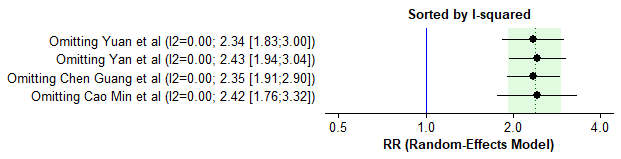


**eFig 20. Forest plots and Leave-one-out analyses for albumin**


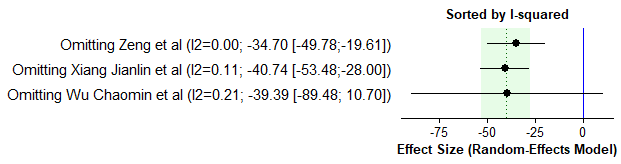

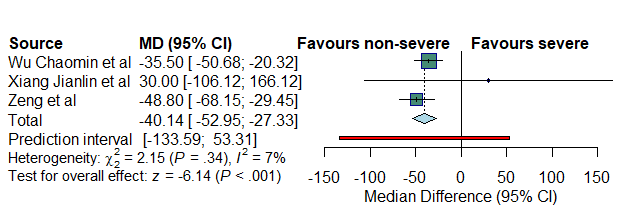


**eFig 21. Forest plot and Leave-one-out analysis for prealbumin**


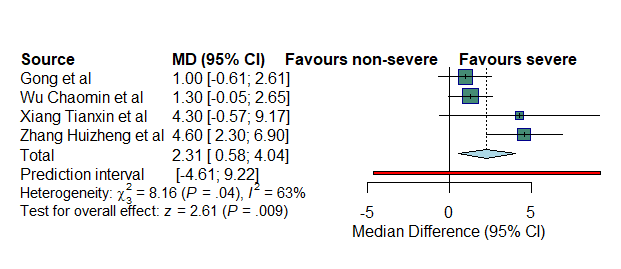

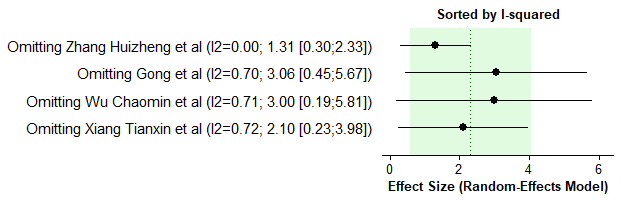


**eFig 22. Forest plot and Leave-one-out analysis for globulin**


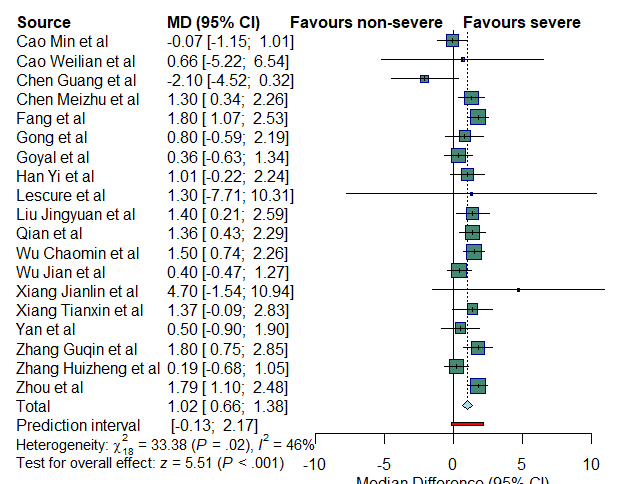

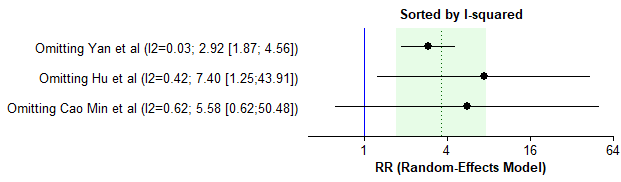

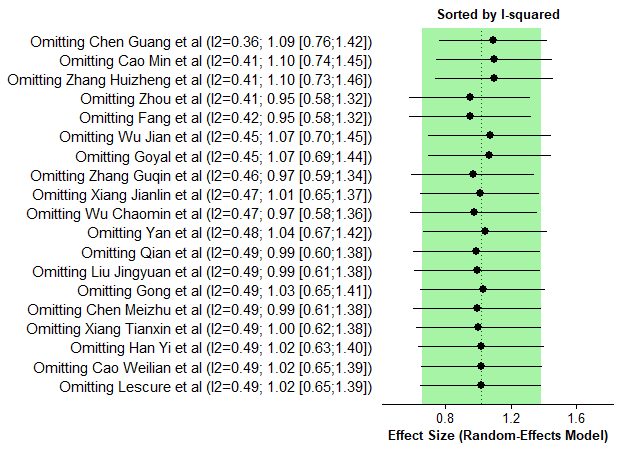

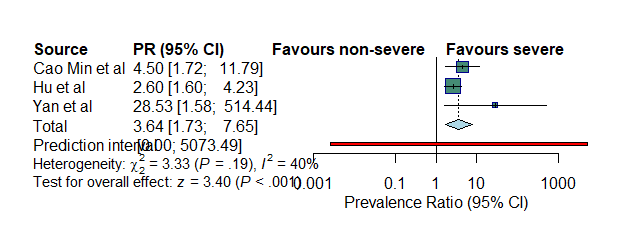


**eFig 23. Forest plots and Leave-one-out analyses for urea**


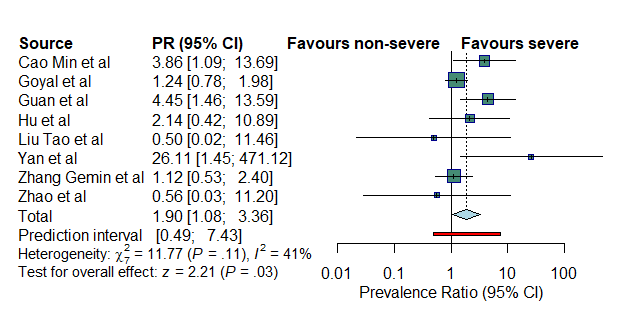

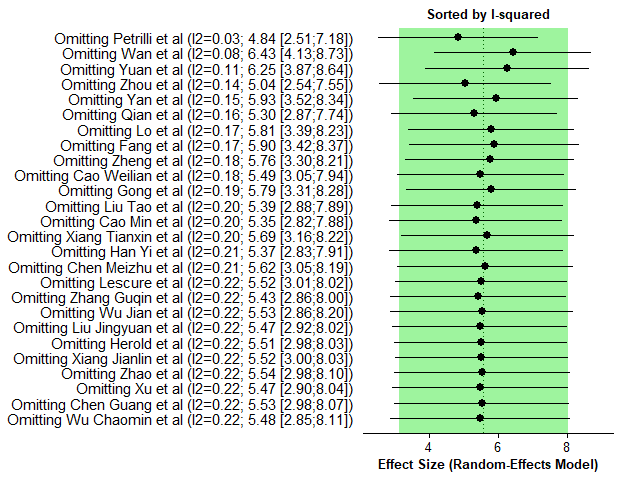

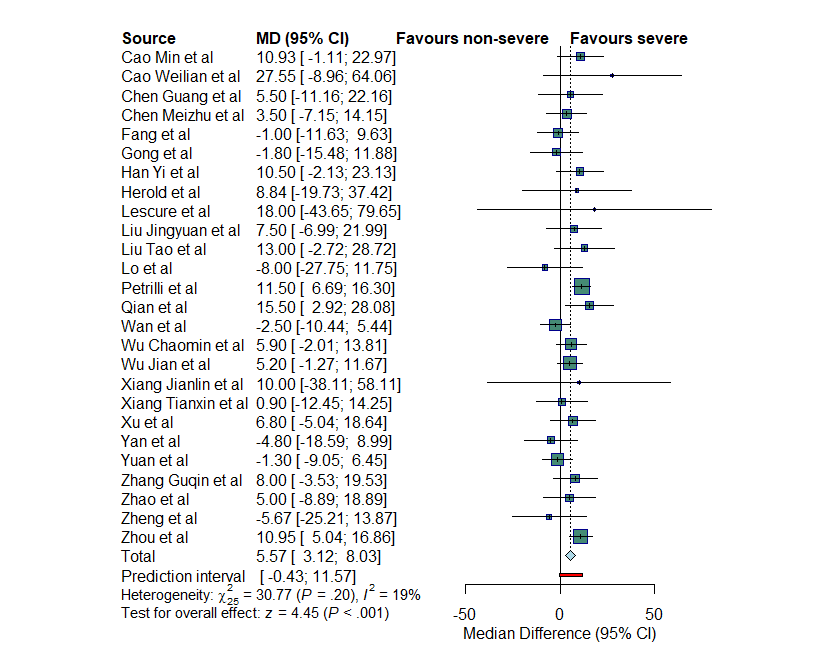

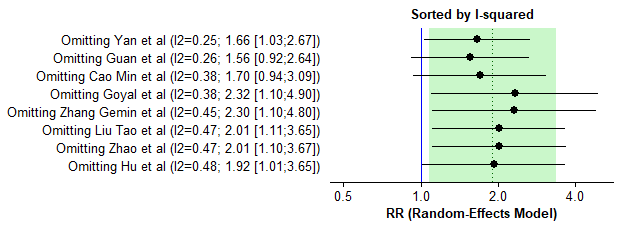


**eFig 24. Forest plots and Leave-one-out analyses for creatinine**


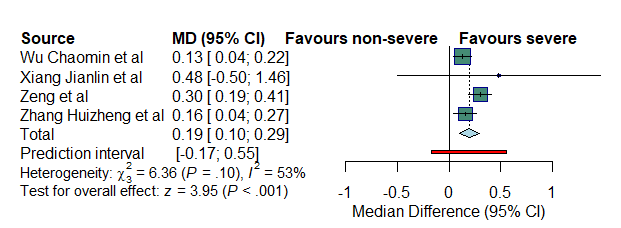

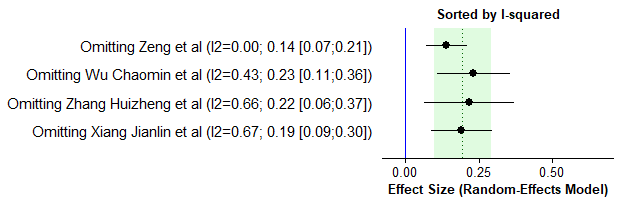


**eFig 25. Forest plot and Leave-one-out analysis for cystatin c**


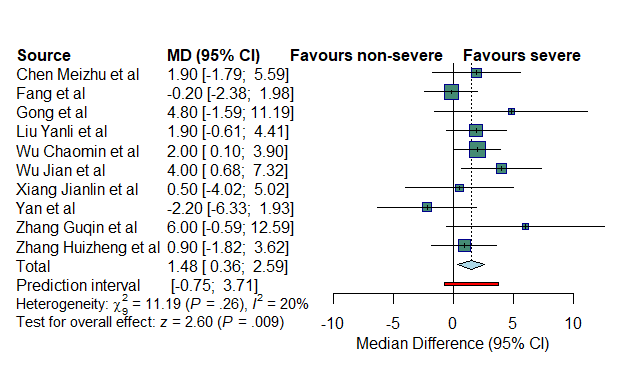

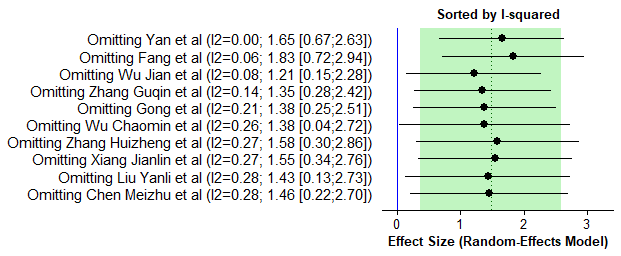


**eFig 26. Forest plot and Leave-one-out analysis for creatine kinase-muscle brain**


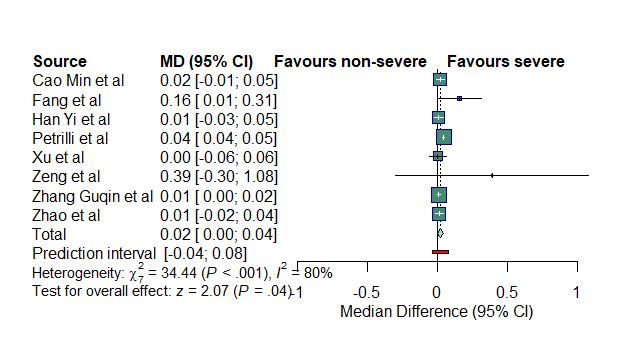

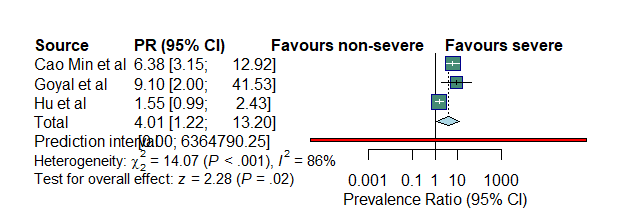

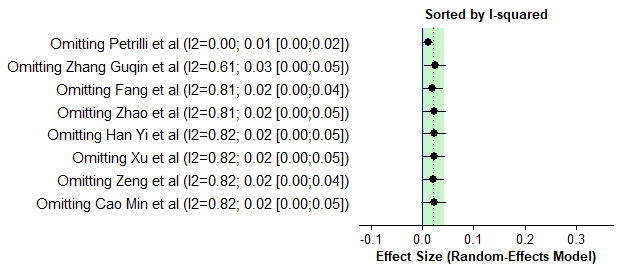

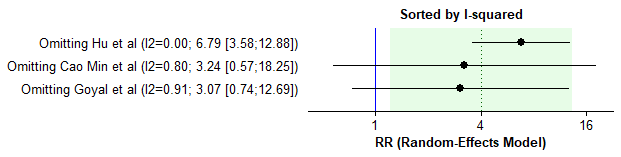


**eFig 27. Forest plots and Leave-one-out analyses for troponin I**


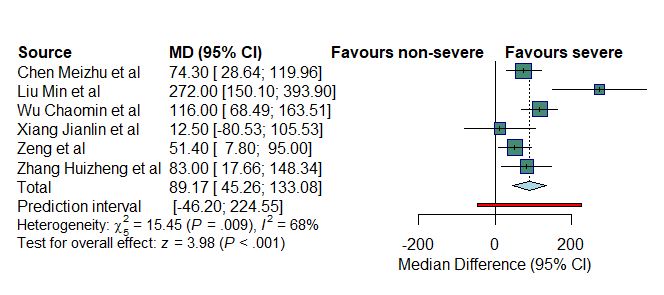

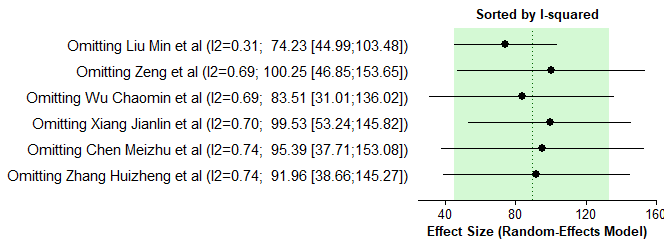


**eFig 28. Forest plot and Leave-one-out analysis for α-hydroxybutyric dehydrogenase**


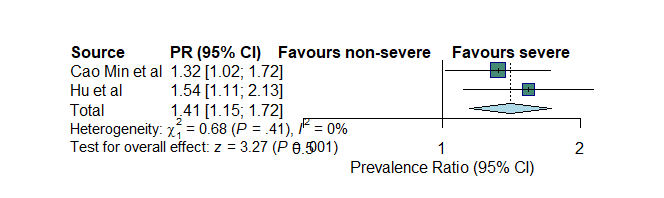

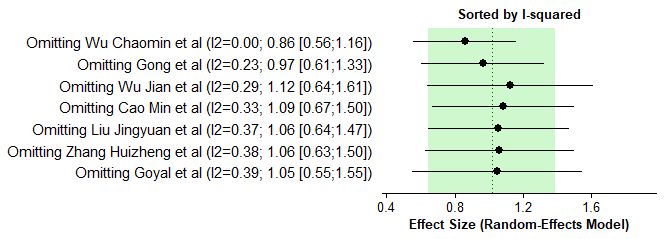

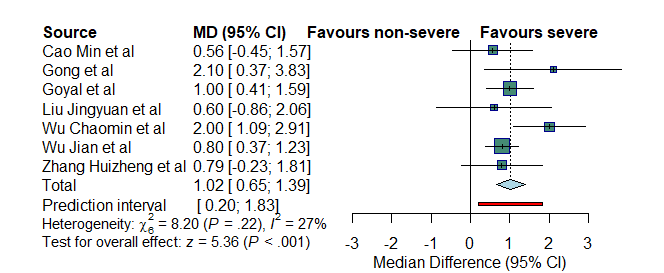

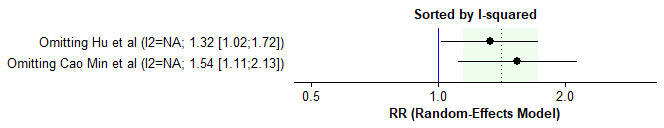


**eFig 29. Forest plots and Leave-one-out analyses for glucose**


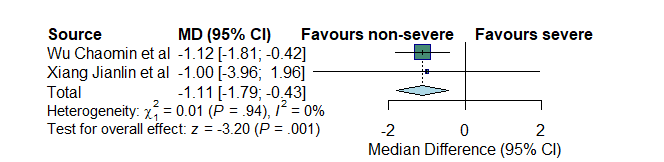

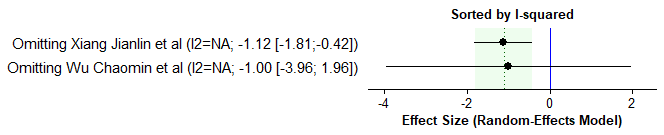


**eFig 30. Forest plot and Leave-one-out analysis for cholinesterase**


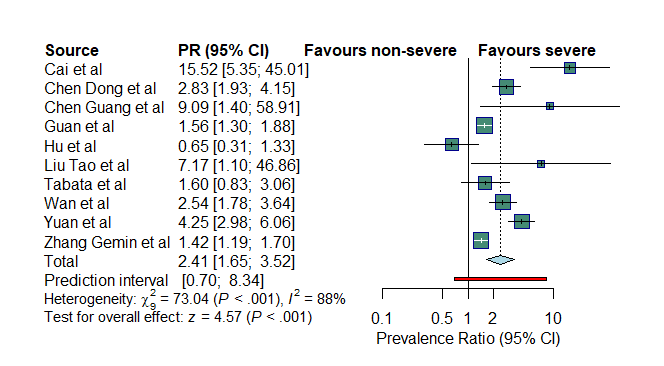

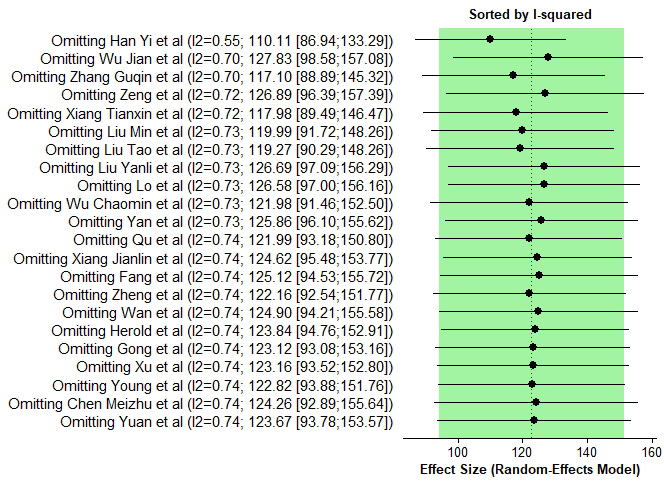

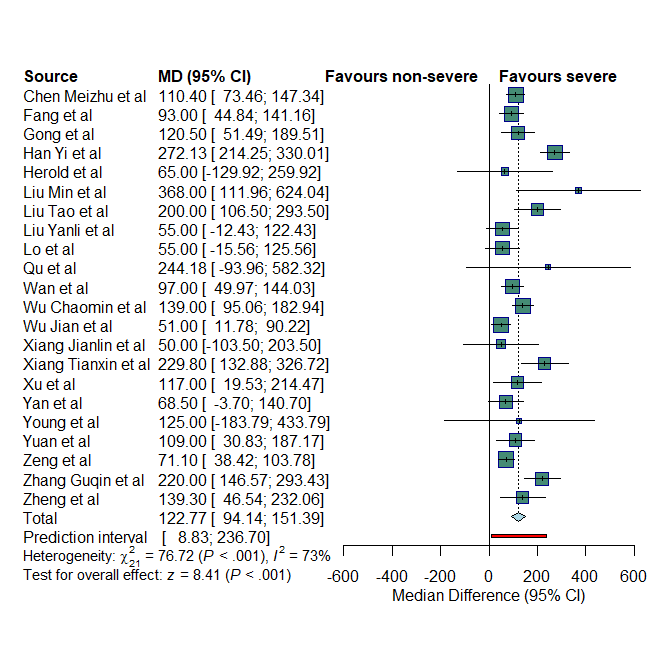

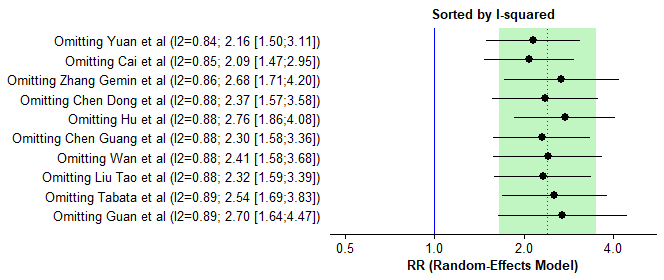


**eFig 31. Forest plots and Leave-one-out analyses for lactate dehydrogenase**


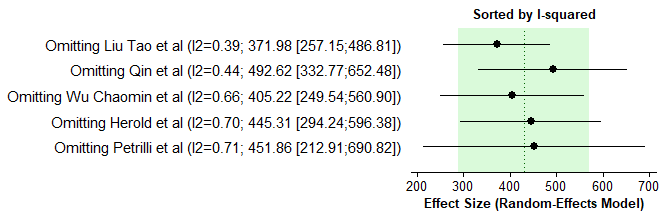

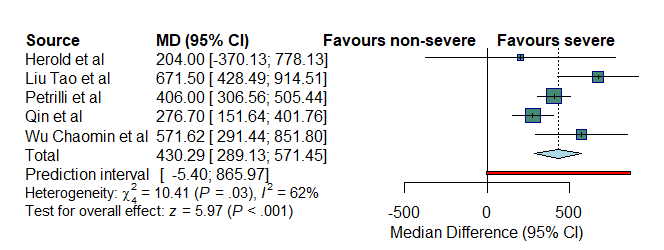


**eFig 32. Forest plots and Leave-one-out analyses for serum ferritin**

**eFig 33. Forest plot and Leave-one-out analysis for sodium**

**eFig 34. Forest plots and Leave-one-out analyses for potassium**

**eFig 35. Forest plot and Leave-one-out analysis for calcium**

**eFig 36. Forest plot and Leave-one-out analysis for chloride**

**Figure 37: Forest plot and Leave-one-out analysis for prothrombin time**

**eFig 38. Forest plot and Leave-one-out analysis for activated partial thromboplastin time**

**Figure 39: Forest plot and Leave-One-Out analysis for D-Dimer**

**eFig 39. Forest plots and Leave-one-out analyses for D-dimer**

# **S5 Table. Risk of bias assessment using NIH Quality Assessment Tool**

| Study ID | q1 | q2 | q3 | q4 | q5 | q6 | q7 | q8 | q9 | q10 | q11 | q12 | q13 | q14 | Overall RoB |  |
| --- | --- | --- | --- | --- | --- | --- | --- | --- | --- | --- | --- | --- | --- | --- | --- | --- |
| Cai et al. | Yes | Yes | Yes | Yes | No | No | No | NA | Yes | No | Yes | NA | NA | No | Low |  |
| Cao Min et al. | Yes | Yes | Yes | Yes | No | No | No | NA | Yes | No | Yes | NA | NA | No | Low |  |
| Cao Weiliang et al. | Yes | Yes | Yes | Yes | No | No | No | NA | No | No | Yes | NA | NA | No | High |  |
| Chen Dong et al. | Yes | Yes | Yes | Yes | No | No | No | NA | Yes | Unclear | Yes | NA | NA | No | Low |  |
| Chen Guang et al. | Yes | Yes | Unclear | Yes | No | No | No | NA | Yes | No | Yes | NA | NA | No | Medium |  |
| Chen Meizhu et al. | Yes | Yes | Yes | Yes | No | No | No | NA | Yes | Yes | Yes | NA | NA | No | Low |  |
| Dai et al. | Yes | Yes | Yes | Yes | No | No | No | NA | Yes | Unclear | Unclear | NA | NA | No | High |  |
| Fang et al. | Yes | Yes | Unclear | Yes | No | No | No | NA | Yes | Unclear | Yes | NA | NA | No | Low |  |
| Gong et al. | Yes | Yes | Yes | Yes | No | No | No | NA | Yes | Yes | Yes | NA | NA | No | Low |  |
| Guan et al. | Yes | Yes | No | Yes | No | No | No | NA | Yes | No | Yes | NA | NA | No | Medium |  |
| Han et al. | Yes | Yes | Unclear | Yes | No | No | No | NA | Yes | No | Yes | NA | NA | No | Low |  |
| Herold et al. | Yes | Yes | Unclear | Yes | No | No | No | NA | Yes | Yes | Yes | NA | NA | No | Low |  |
| Hu et al. | Yes | Yes | Unclear | Yes | No | No | No | NA | Yes | No | Yes | NA | NA | No | Medium |  |
| Liu Chuan et al. | Yes | Yes | Unclear | Yes | No | No | No | NA | Yes | Unclear | Yes | NA | NA | No | High |  |
| Liu Jingyuan et al. | Yes | Yes | Unclear | Yes | No | No | No | NA | Yes | No | Yes | NA | NA | No | Low |  |
| Liu Min et al. | Yes | Yes | Yes | Yes | No | No | No | NA | Yes | Unclear | Yes | NA | NA | No | Medium |  |
| Liu Tao et al. | Yes | Yes | Unclear | Yes | No | No | No | NA | Yes | No | Yes | NA | NA | No | Medium |  |
| Liu Yanli et al. | Yes | Yes | Unclear | Yes | No | No | No | NA | Yes | Unclear | Yes | NA | NA | No | Medium |  |
| Luo et al. | Yes | Yes | Yes | Yes | No | No | No | NA | Yes | No | Yes | NA | NA | No | Low |  |
| Petrilli et al. | Yes | Yes | Yes | Yes | No | No | No | NA | Yes | Yes | Yes | NA | NA | No | Low |  |
| Qian et al. | Yes | Yes | Yes | No | No | No | No | NA | Yes | No | Yes | NA | NA | No | Medium |  |
| Qin et al. | Yes | Yes | Unclear | Yes | No | No | No | NA | Yes | No | Yes | NA | NA | No | Low |  |
| Qu et al. | Yes | Yes | Unclear | Yes | No | No | No | NA | Yes | No | Yes | NA | NA | No | Low |  |
| Tabata et al. | Yes | Yes | Yes | Yes | No | No | No | NA | Yes | Yes | Yes | NA | NA | No | Low |  |
| Wu Chaomin et al. | Yes | Yes | Unclear | Yes | No | No | No | NA | Yes | Yes | Yes | NA | NA | No | Low |  |
| Wu Jian et al. | Yes | Yes | Unclear | Yes | No | No | No | NA | Yes | Unclear | Yes | NA | NA | No | Medium |  |
| Xiang Jialin et al. | Yes | Yes | Unclear | Yes | No | No | No | NA | Yes | Unclear | Yes | NA | NA | No | Medium |  |
| Xiang Tianxin et al. | Yes | Yes | Yes | Yes | No | No | No | NA | Yes | No | Yes | NA | NA | No | Low |  |
| Yan et al. | Yes | Yes | Unclear | Yes | No | No | No | NA | Yes | Unclear | Yes | NA | NA | No | Medium |  |
| Yuan et al. | Yes | Yes | Yes | Yes | No | No | No | NA | Yes | No | Yes | NA | NA | No | Low |  |
| Zeng et al. | Yes | Yes | Unclear | Yes | No | No | No | NA | Yes | Yes | Yes | NA | NA | No | Low |  |
| Zhang Gemin et al. | Yes | Yes | Unclear | Yes | No | No | No | NA | Yes | Unclear | Yes | NA | NA | No | Low |  |
| Zhang Guqin et al. | Yes | Yes | Unclear | Yes | No | No | No | NA | Yes | No | Yes | NA | NA | No | Low |  |
| Zhang Huizheng et al. | Yes | Yes | Unclear | Yes | No | No | No | NA | Yes | No | Yes | NA | NA | No | Medium |  |
| Zhang Jin-jin et al. | Yes | Yes | Yes | Yes | No | No | No | NA | Yes | No | Yes | NA | NA | No | Medium |  |
| Zhao et al. | Yes | Yes | Yes | Yes | No | No | No | NA | Yes | No | Yes | NA | NA | No | Low |  |
| Zheng et al. | No | Yes | Unclear | Yes | No | No | No | NA | Yes | Unclear | Yes | NA | NA | No | Medium |  |
| Zhou et al. | Yes | Yes | Unclear | Yes | No | No | No | NA | Yes | No | Yes | NA | NA | No | Low |  |
| Case Series Studies | | | | | | | | | | | | | | | | |
| Goyal et al. | Yes | Yes | Yes | Yes | Yes | Yes | NA | Yes | Yes |  |  |  |  |  | Low |  |
| Lescure et al. | Yes | Yes | Yes | Yes | Yes | Yes | NA | Yes | Yes |  |  |  |  |  | Low |  |
| Lo et al. | Yes | Yes | Yes | Yes | Yes | Yes | NA | Yes | Yes |  |  |  |  |  | Low |  |
| Wan et al. | Yes | Yes | Yes | Yes | Yes | Yes | NA | Yes | Yes |  |  |  |  |  | Low |  |
| Wang et al. | Yes | Yes | Yes | Yes | Yes | Yes | NA | Yes | Yes |  |  |  |  |  | Low |  |
| Xu et al. | Yes | Yes | Yes | Yes | Yes | Yes | NA | Yes | Yes |  |  |  |  |  | Low |  |
| Young et al. | Yes | Yes | Yes | Yes | Yes | Yes | NA | No | Yes |  |  |  |  |  | Medium |  |

NA: Not applicable

**For cohort and cross-sectional studies:**

q1: Was the research question or objective in this paper clearly stated?

q2: Was the study population clearly specified and defined?

q3: Was the participation rate of eligible persons at least 50%?

q4: Were all the subjects selected or recruited from the same or similar populations (including the same time period)? Were inclusion and exclusion criteria for being in the study prespecified and applied uniformly to all participants?

q5: Was a sample size justification, power description, or variance and effect estimates provided?

q6: For the analyses in this paper, were the exposure(s) of interest measured prior to the outcome(s) being measured?

q7: Was the timeframe sufficient so that one could reasonably expect to see an association between exposure and outcome if it existed?

q8: For exposures that can vary in amount or level, did the study examine different levels of the exposure as related to the outcome (e.g., categories of exposure, or exposure measured as continuous variable)?

q9: Were the exposure measures (independent variables) clearly defined, valid, reliable, and implemented consistently across all study participants?

q10: Was the exposure(s) assessed more than once over time?

q11: Were the outcome measures (dependent variables) clearly defined, valid, reliable, and implemented consistently across all study participants?

q12: Were the outcome assessors blinded to the exposure status of participants?

q13: Was loss to follow-up after baseline 20% or less?

q14: Were key potential confounding variables measured and adjusted statistically for their impact on the relationship between exposure(s) and outcome(s)?

**For case series:**

q1: Was the study question or objective clearly stated?

q2: Was the study population clearly and fully described, including a case definition?

q3: Were the cases consecutive?

q4: Were the subjects comparable?

q5: Was the intervention clearly described?

q6: Were the outcome measures clearly defined, valid, reliable, and implemented consistently across all study participants?

q7: Was the length of follow-up adequate?

q8: Were the statistical methods well-described?

q9: Were the results well-described?

q10: Was the exposure(s) assessed more than once over time?

# **S6 Table. Sensitivity analysis reporting mean differences obtained by estimating the sample mean and variance from the reported median and interquartile range (or range)**

| Parameter | Number of studies | Number of persons | Meta-mean difference (95% CI) | *p* value | Prediction interval | I^2^ | Q test p value |
| --- | --- | --- | --- | --- | --- | --- | --- |
| Hematological parameters | |  |  |  |  |  |  |
| White cell count, ×10^9^/L | 28 | 4749 | 1.28 (0.85, 1.71) | <0.001 | -0.97, 3.53 | 99.8% | 0.000 |
| Neutrophils, ×10^9^/L | 21 | 3091 | 1.71 (0.98, 2.43) | <0.001 | -1.84, 5.25 | 99.9% | 0.000 |
| Lymphocytes, ×10^9^/L | 29 | 6465 | -0.39 (-0.45, -0.32) | <0.001 | -0.72, -0.05 | 99.9% | 0.000 |
| Monocytes, ×10^9^/L | 14 | 2002 | -0.01 (-0.03, 0.01) | 0.415 | -0.09, 0.07 | 98.4% | <0.001 |
| Platelets, ×10^9^/L | 24 | 3877 | -17.02 (-25.17, -8.88) | <0.001 | -56.38, 22.33 | 99.0% | 0.000 |
| Hemoglobin, g/dl | 17 | 2931 | -0.36 (-0.66, -0.05) | 0.0206 | -1.61, 0.90 | 98.9% | <0.001 |
| CD3, cells/μl | 6 | 601 | -392.38 (-520.17, -264.58) | <0.001 | -845.66, 60.91 | 98.8% | <0.001 |
| CD4, cells/μl | 7 | 669 | -194.87 (-286.64, -103.11) | <0.001 | -523.19, 133.45 | 99.3% | <0.001 |
| CD8, cells/μl | 6 | 600 | -134.17 (-192.22, -76.12) | <0.001 | -338.45, 70.11 | 99.2% | <0.001 |
| NLR | 5 | 1377 | 2.60 (2.19, 3.02) | <0.001 | 0.97, 4.23 | 99.6% | <0.001 |
| SII | 2 | 487 | 396.89 (117.16, 676.61) | 0.005 | - | 99.5% | <0.001 |
| Infection/inflammation-related indices | | |  |  |  |  |  |
| C-reactive protein, mg/L | 26 | 4959 | 40.36 (27.90, 52.82) | <0.001 | -26.40, 107.12 | 100% | 0.000 |
| ESR, mm/hr | 8 | 1705 | 16.34 (10.24, 22.44) | <0.001 | -4.29, 36.97 | 99.7% | 0.000 |
| Interleukin-6, pg/ml | 7 | 1183 | 31.27 (17.12, 45.42) | <0.001 | -20.48, 83.02 | 100% | 0.000 |
| Procalcitonin, ng/ml | 16 | 4225 | 0.13 (0.07, 0.18) | <0.001 | -0.11, 0.36 | 100% | 0.000 |
| Liver function parameters |  |  |  |  |  |  |  |
| ALT, U/L | 25 | 4450 | 8.11 (6.73, 9.48) | <0.001 | 1.99, 14.21 | 98.6% | 0.000 |
| AST, U/L | 25 | 4320 | 14.64 (12.17, 17.12) | <0.001 | 3.05, 26.24 | 99.6% | 0.000 |
| Total bilirubin | 18 | 2104 | 1.92 (0.96, 2.87) | <0.001 | -2.17, 6.00 | 98.9% | <0.001 |
| Total protein, g/L | 5 | 482 | 1.69 (-0.68, 4.06) | 0.162 | -6.72, 10.09 | 97.4% | <0.001 |
| Albumin, g/L | 21 | 2891 | -4.50 (-5.99, -3.01) | <0.001 | -11.64, 2.64 | 99.9% | 0.000 |
| Prealbumin, mg/dl | 3 | 367 | -18.40 (-50.80, 13.99) | 0.265 | -416.87, 380.06 | 90.8% | <0.001 |
| Globulin, g/L | 4 | 476 | 2.54 (1.41, 3.67) | <0.001 | -2.50, 7.58 | 97.9% | <0.001 |
| Kidney function parameters | |  |  |  |  |  |  |
| Blood urea, mmol/l | 19 | 2623 | 1.07 (0.70, 1.43) | <0.001 | -0.51, 2.64 | 99.2% | 0.000 |
| Creatinine, μmol/l | 26 | 4467 | 7.20 (4.55, 9.85) | <0.001 | -6.03, 20.43 | 99.4% | 0.000 |
| Cystatin C, mg/L | 4 | 426 | 0.22 (0.14, 0.31) | <0.001 | -0.13, 0.58 | 95.6% | <0.001 |
| Myocardial biomarkers |  |  |  |  |  |  |  |
| CK-MB, U/L | 10 | 1324 | 2.19 (0.81, 3.57) | 0.002 | -2.98, 29.18 | 99.1% | <0.001 |
| Troponin I, ng/ml | 8 | 2379 | 0.04 (0.03, 0.04) | <0.001 | 0.02, 0.06 | 98.0% | <0.001 |
| α-HBDH, U/L | 6 | 465 | 87.25 (49.33, 125.18) | <0.001 | -44.49, 219.00 | 98.1% | <0.001 |
| Other biochemical parameters | | |  |  |  |  |  |
| Glucose, mmol/L | 7 | 1343 | 1.21 (0.75, 1.67) | <0.001 | -0.40, 2.81 | 99.6% | <0.001 |
| Cholinesterase, U/ml | 2 | 229 | -1.17 (-1.59, -0.75) | <0.001 | - | 51.5% | 0.151 |
| LDH, U/L | 22 | 2297 | 137.51 (111.82, 163.21) | <0.001 | 16.00, 259.03 | 99.4% | 0.000 |
| Serum ferritin, μg/L | 5 | 2342 | 466.70 (391.16, 542.24) | <0.001 | 198.25, 735.14 | 99.5% | <0.001 |
| Serum electrolytes |  |  |  |  |  |  |  |
| Sodium, mmol/L) | 10 | 1503 | -2.16 (-3.30, -1.02) | <0.001 | -6.33, 2.00 | 99.2% | <0.001 |
| Potassium, mmol/L) | 12 | 1790 | -0.16 (-0.21, 0.11) | <0.001 | -0.35, 0.03 | 95.7% | <0.001 |
| Chloride, mmol/L) | 6 | 1074 | -1.23 (-2.61, 0.15) | 0.080 | -6.03, 3.56 | 98.5% | <0.001 |
| Calcium, mmol/L) | 5 | 486 | -0.13 (-0.18, -0.08) | <0.001 | -0.32, 0.05 | 97.5% | <0.001 |
| Coagulation parameters |  |  |  |  |  |  |  |
| Prothrombin time, s | 16 | 1650 | 0.42 (0.14, 0.70) | 0.003 | -0.76, 1.60 | 99.7% | 0.000 |
| APTT, s | 14 | 1918 | -0.34 (-1.54, 0.86) | 0.577 | -5.20, 4.51 | 98.7% | <0.001 |
| D-dimer, mg/L | 23 | 4740 | 1.30 (1.12, 1.47) | <0.001 | 0.46, 2.14 | 99.9% | 0.000 |

NLR: neutrophil-to-lymphocyte ratio; SII: systemic inflammation Index; ALT: Alanine aminotransferase; AST: Aspartate aminotransferase; LDH: Lactate Dehydrogenase; APTT: Activated partial thromboplastin time

# **References for supplementary file**

1. Cai Q, Huang D, Ou P, Yu H, Zhu Z, Xia Z, et al. COVID-19 in a Designated Infectious Diseases HospitalOutside Hubei Province,China. medRxiv. 2020:2020.02.17.20024018. doi: 10.1101/2020.02.17.20024018.

2. Cao M, Zhang D, Wang Y, Lu Y, Zhu X, Li Y, et al. Clinical Features of Patients Infected with the 2019 Novel Coronavirus (COVID-19) in Shanghai, China. medRxiv. 2020:2020.03.04.20030395. doi: 10.1101/2020.03.04.20030395.

3. Cao W. Clinical features and laboratory inspection of novel coronavirus pneumonia (COVID-19) in Xiangyang, Hubei. medRxiv. 2020:2020.02.23.20026963. doi: 10.1101/2020.02.23.20026963.

4. chen d, Li X, song q, Hu C, Su F, Dai J. Hypokalemia and Clinical Implications in Patients with Coronavirus Disease 2019 (COVID-19). medRxiv. 2020:2020.02.27.20028530. doi: 10.1101/2020.02.27.20028530.

5. Chen G, Wu D, Guo W, Cao Y, Huang D, Wang H, et al. Clinical and immunologic features in severe and moderate Coronavirus Disease 2019. The Journal of clinical investigation. 2020;27. PubMed PMID: 631358781.

6. chen m, tu c, Tan C, Zheng X, wang x, wu j, et al. Key to successful treatment of COVID-19: accurate identification of severe risks and early intervention of disease progression. medRxiv. 2020:2020.04.06.20054890. doi: 10.1101/2020.04.06.20054890.

7. Dai Z, Gao L, Luo D, Xiao J, Huang C, Zeng G, et al. [Analysis of clinical characteristics of new coronavirus pneumonia in Hunan Province]. Practical Preventive Medicine. 2020:1-4.

8. Fang X, Mei Q, Yang T, Zhang L, Yang Y, Wang Y, et al. [2019 New Coronavirus Infected Pneumonia: Clinical Features and Treatment Analysis of 79 Cases]. Chinese Pharmacology Bulletin. 2020;36(4):1-7.

9. Gong J, Ou J, Qiu X, Jie Y, Chen Y, Yuan L, et al. A Tool to Early Predict Severe 2019-Novel Coronavirus Pneumonia (COVID-19) : A Multicenter Study using the Risk Nomogram in Wuhan and Guangdong, China. medRxiv. 2020:2020.03.17.20037515. doi: 10.1101/2020.03.17.20037515.

10. Goyal P, Choi JJ, Pinheiro LC, Schenck EJ, Chen R, Jabri A, et al. Clinical Characteristics of Covid-19 in New York City. The New England journal of medicine. 2020. doi: <https://dx.doi.org/10.1056/NEJMc2010419>.

11. Guan WJ, Ni ZY, Hu Y, Liang WH, Ou CQ, He JX, et al. Clinical Characteristics of Coronavirus Disease 2019 in China. New England Journal of Medicine. 2020;28:28. PubMed PMID: 32109013.

12. Han Y, Zhang H, Mu S, Wei W, Jin C, Xue Y, et al. Lactate dehydrogenase, a Risk Factor of Severe COVID-19 Patients. medRxiv. 2020:2020.03.24.20040162. doi: 10.1101/2020.03.24.20040162.

13. Herold T, Jurinovic V, Arnreich C, Hellmuth JC, Bergwelt-Baildon M, Klein M, et al. Level of IL-6 predicts respiratory failure in hospitalized symptomatic COVID-19 patients. medRxiv. 2020:2020.04.01.20047381. doi: 10.1101/2020.04.01.20047381.

14. Hu L, Chen S, Fu Y, Gao Z, Long H, Ren H-w, et al. Risk Factors Associated with Clinical Outcomes in 323 COVID-19 Patients in Wuhan, China. medRxiv. 2020:2020.03.25.20037721. doi: 10.1101/2020.03.25.20037721.

15. Lescure FX, Bouadma L, Nguyen D, Parisey M, Wicky PH, Behillil S, et al. Clinical and virological data of the first cases of COVID-19 in Europe: a case series. The Lancet Infectious Diseases. 2020;27:27. PubMed PMID: 32224310.

16. Liu C, Jiang ZC, Shao CX, Zhang HG, Yue HM, Chen ZH, et al. Preliminary study of the relationship between novel coronavirus pneumonia and liver function damage: a multicenter study. [Chinese]. Zhonghua gan zang bing za zhi = Zhonghua ganzangbing zazhi = Chinese journal of hepatology. 2020;28(2):148-52. PubMed PMID: 631012069.

17. Liu J, Liu Y, Xiang P, Pu L, Xiong H, Li C, et al. Neutrophil-to-Lymphocyte Ratio Predicts Severe Illness Patients with 2019 Novel Coronavirus in the Early Stage. medRxiv. 2020:2020.02.10.20021584. doi: 10.1101/2020.02.10.20021584.

18. Liu M, He P, Liu HG, Wang XJ, Li FJ, Chen S, et al. [Clinical characteristics of 30 medical workers infected with new coronavirus pneumonia]. Chung-Hua Chieh Ho Ho Hu Hsi Tsa Chih Chinese Journal of Tuberculosis & Respiratory Diseases. 2020;43(3):209-14. PubMed PMID: 32164090.

19. Liu T, Zhang J, Yang Y, Ma H, Li Z, Zhang J, et al. The potential role of IL-6 in monitoring severe case of coronavirus disease 2019. medRxiv. 2020:2020.03.01.20029769. doi: 10.1101/2020.03.01.20029769.

20. Liu Y, Sun W, Li J, Chen L, Wang Y, Zhang L, et al. Clinical features and progression of acute respiratory distress syndrome in coronavirus disease 2019. medRxiv. 2020:2020.02.17.20024166. doi: 10.1101/2020.02.17.20024166.

21. Lo IL, Lio CF, Cheong HH, Lei CI, Cheong TH, Zhong X, et al. Evaluation of SARS-CoV-2 RNA shedding in clinical specimens and clinical characteristics of 10 patients with COVID-19 in Macau. International Journal of Biological Sciences [Electronic Resource]. 2020;16(10):1698-707. PubMed PMID: 32226287.

22. Luo X, Zhou W, Yan X, Guo T, Wang B, Xia H, et al. Prognostic value of C-reactive protein in patients with COVID-19. medRxiv. 2020:2020.03.21.20040360. doi: 10.1101/2020.03.21.20040360.

23. Petrilli CM, Jones SA, Yang J, Rajagopalan H, Donnell LF, Chernyak Y, et al. Factors associated with hospitalization and critical illness among 4,103 patients with COVID-19 disease in New York City. medRxiv. 2020:2020.04.08.20057794. doi: 10.1101/2020.04.08.20057794.

24. Qian G-Q, Yang N-B, Ding F, Ma AHY, Wang Z-Y, Shen Y-F, et al. Epidemiologic and Clinical Characteristics of 91 Hospitalized Patients with COVID-19 in Zhejiang, China: A retrospective, multi-centre case series. medRxiv. 2020:2020.02.23.20026856. doi: 10.1101/2020.02.23.20026856.

25. Qin C, Zhou L, Hu Z, Zhang S, Yang S, Tao Y, et al. Dysregulation of immune response in patients with COVID-19 in Wuhan, China. Clinical Infectious Diseases. 2020;12:12. PubMed PMID: 32161940.

26. Qu R, Ling Y, Zhang YH, Wei LY, Chen X, Li XM, et al. Platelet-to-lymphocyte ratio is associated with prognosis in patients with coronavirus disease-19. Journal of Medical Virology. 2020;17:17. PubMed PMID: 32181903.

27. Tabata S, Imai K, Kawano S, Ikeda M, Kodama T, Miyoshi K, et al. Non-severe vs severe symptomatic COVID-19: 104 cases from the outbreak on the cruise ship “Diamond Princess” in Japan. medRxiv. 2020:2020.03.18.20038125. doi: 10.1101/2020.03.18.20038125.

28. Wan S, Xiang Y, Fang W, Zheng Y, Li B, Hu Y, et al. Clinical Features and Treatment of COVID-19 Patients in Northeast Chongqing. Journal of Medical Virology. 2020;21:21. PubMed PMID: 32198776.

29. Wang Z, Yang B, Li Q, Wen L, Zhang R. Clinical Features of 69 Cases with Coronavirus Disease 2019 in Wuhan, China. Clinical Infectious Diseases. 2020;16:16. PubMed PMID: 32176772.

30. Wu C, Chen X, Cai Y, Xia J, Zhou X, Xu S, et al. Risk Factors Associated With Acute Respiratory Distress Syndrome and Death in Patients With Coronavirus Disease 2019 Pneumonia in Wuhan, China. JAMA Internal Medicine. 2020;13:13. PubMed PMID: 32167524.

31. Wu J, Li W, Shi X, Chen Z, Jiang B, Liu J, et al. Early antiviral treatment contributes to alleviate the severity and improve the prognosis of patients with novel coronavirus disease (COVID-19). Journal of Internal Medicine. 2020;27:27. PubMed PMID: 32220033.

32. Xiang J, Wen J, Yuan X, Xiong S, Zhou XUE, Liu C, et al. Potential biochemical markers to identify severe cases among COVID-19 patients. medRxiv. 2020:2020.03.19.20034447. doi: 10.1101/2020.03.19.20034447.

33. Xiang T, Liu J, Xu F, Cheng N, Liu Y, Qian K, et al. [Clinical characteristics of 49 patients with novel coronavirus pneumonia in Jiangxi area]. Chinese Journal of Respiratory and Critical Care Medicine. 2020:1-7.

34. Xu Y, Li Y-r, Zeng Q, Lu Z-b, Li Y-z, Wu W, et al. Clinical Characteristics of SARS-CoV-2 Pneumonia Compared to Controls in Chinese Han Population. medRxiv. 2020:2020.03.08.20031658. doi: 10.1101/2020.03.08.20031658.

35. Yan S, Song X, Lin F, Zhu H, Wang X, Li M, et al. Clinical Characteristics of Coronavirus Disease 2019 in Hainan, China. medRxiv. 2020:2020.03.19.20038539. doi: 10.1101/2020.03.19.20038539.

36. Young BE, Ong SWX, Kalimuddin S, Low JG, Tan SY, Loh J, et al. Epidemiologic Features and Clinical Course of Patients Infected With SARS-CoV-2 in Singapore. Jama. 2020;03:03. PubMed PMID: 32125362.

37. Yuan J, Sun Y, Zuo Y, Chen T, Cao Q, Yuan G, et al. [Clinical characteristics of 223 patients with new coronavirus pneumonia in Chongqing]. Journal of Southwest University (Natural Science Edition). 2020:1-7.

38. Zeng L, Li J, Liao M, Hua R, Huang P, Zhang M, et al. Risk assessment of progression to severe conditions for patients with COVID-19 pneumonia: a single-center retrospective study. medRxiv. 2020:2020.03.25.20043166. doi: 10.1101/2020.03.25.20043166.

39. Zhang G, Zhang J, Wang B, Zhu X, Wang Q, Qiu S. Analysis of clinical characteristics and laboratory findings of 95 cases of 2019 novel coronavirus pneumonia in Wuhan, China: a retrospective analysis. Respiratory Research. 2020;21(1):74. PubMed PMID: 32216803.

40. Zhang G, Hu C, Luo L, Fang F, Chen Y, Li J, et al. Clinical features and outcomes of 221 patients with COVID-19 in Wuhan, China. medRxiv. 2020:2020.03.02.20030452. doi: 10.1101/2020.03.02.20030452.

41. zhang h, wang x, fu z, luo m, zhang z, zhang k, et al. Potential Factors for Prediction of Disease Severity of COVID-19 Patients. medRxiv. 2020:2020.03.20.20039818. doi: 10.1101/2020.03.20.20039818.

42. Zhang JJ, Dong X, Cao YY, Yuan YD, Yang YB, Yan YQ, et al. Clinical characteristics of 140 patients infected with SARS-CoV-2 in Wuhan, China. Allergy. 2020;19:19. PubMed PMID: 32077115.

43. Zhao W, Yu S, Zha X, Wang N, Pang Q, Li T, et al. Clinical characteristics and durations of hospitalized patients with COVID-19 in Beijing: a retrospective cohort study. medRxiv. 2020:2020.03.13.20035436. doi: 10.1101/2020.03.13.20035436.

44. Zheng M, Gao Y, Wang G, Song G, Liu S, Sun D, et al. Functional exhaustion of antiviral lymphocytes in COVID-19 patients. Cellular & Molecular Immunology. 2020;19:19. PubMed PMID: 32203188.

45. Zhou Y, Yang Z, Guo Y, Geng S, Gao S, Ye S, et al. A New Predictor of Disease Severity in Patients with COVID-19 in Wuhan, China. medRxiv. 2020:2020.03.24.20042119. doi: 10.1101/2020.03.24.20042119.
