# Supplementary material for: Clinical laboratory parameters associated with severe or critical novel coronavirus disease 2019 (COVID-19): A systematic review and meta-analysis
Source: PLoS One. 2020 Oct 1;15(10):e0239802. doi: 10.1371/journal.pone.0239802 (PMC7529271; doi:10.1371/journal.pone.0239802)
Supplement: Peer review history

# Editor report: Decision Letter 0

**DOI:** 10.1371/journal.pone.0239802.r001

**Authors:** Anna Simonin

15 Sep 2020

Clinical laboratory parameters associated with severe or critical novel coronavirus disease 2019 (COVID-19): a systematic review and meta-analysis

PONE-D-20-24329

Dear Dr. Moutchia,

We’re pleased to inform you that your manuscript has been judged scientifically suitable for publication and will be formally accepted for publication once it meets all outstanding technical requirements.

Within one week, you’ll receive an e-mail detailing the required amendments. When these have been addressed, you’ll receive a formal acceptance letter and your manuscript will be scheduled for publication.

An invoice for payment will follow shortly after the formal acceptance. To ensure an efficient process, please log into Editorial Manager at http://www.editorialmanager.com/pone/, click the 'Update My Information' link at the top of the page, and double check that your user information is up-to-date. If you have any billing related questions, please contact our Author Billing department directly at authorbilling@plos.org.

If your institution or institutions have a press office, please notify them about your upcoming paper to help maximize its impact. If they’ll be preparing press materials, please inform our press team as soon as possible -- no later than 48 hours after receiving the formal acceptance. Your manuscript will remain under strict press embargo until 2 pm Eastern Time on the date of publication. For more information, please contact onepress@plos.org.

Kind regards,

Anna Simonin, Ph.D.

Senior Editor

PLOS ONE

Additional Editor Comments (optional):

Since your manuscript was previously and thoroughly reviewed and this is a resubmission of the same manuscript, we are accepting your manuscript without further review.  You have addressed all the concerns raised by the previous reviewers and Academic Editor. In addition, the Academic Editor has confirmed that your manuscript is ready to be published. Thank you for your patience! 

Reviewers' comments:

# Editor report: Acceptance letter

**DOI:** 10.1371/journal.pone.0239802.r002

**Authors:** Anna Simonin

22 Sep 2020

PONE-D-20-24329 

Clinical laboratory parameters associated with severe or critical novel coronavirus disease 2019 (COVID-19): a systematic review and meta-analysis 

Dear Dr. Moutchia:

I'm pleased to inform you that your manuscript has been deemed suitable for publication in PLOS ONE. Congratulations! Your manuscript is now with our production department. 

If your institution or institutions have a press office, please let them know about your upcoming paper now to help maximize its impact. If they'll be preparing press materials, please inform our press team within the next 48 hours. Your manuscript will remain under strict press embargo until 2 pm Eastern Time on the date of publication. For more information please contact onepress@plos.org.

If we can help with anything else, please email us at plosone@plos.org. 

Thank you for submitting your work to PLOS ONE and supporting open access. 

Kind regards, 

PLOS ONE Editorial Office Staff

on behalf of

Dr. Anna Simonin 

%CORR_ED_EDITOR_ROLE%

PLOS ONE
